# Evaluating density-weighted connectivity of black bears (*Ursus americanus*) in Glacier National Park with spatial capture–recapture models

**DOI:** 10.1186/s40462-023-00445-7

**Published:** 2024-01-23

**Authors:** Sarah L. Carroll, Greta M. Schmidt, John S. Waller, Tabitha A. Graves

**Affiliations:** 1 Graduate Degree Program in Ecology, Colorado State University, Fort Collins, CO 80523 USA; 2 Department of Biology, San Diego State University, San Diego, CA 92182 USA; 3 Glacier National Park, P.O. Box 128, West Glacier, MT 59936 USA; 4 U.S. Geological Survey, Northern Rocky Mountain Science Center, PO Box 169, West Glacier, MT 59936 USA

**Keywords:** Spatial capture–recapture, Density, Movement connectivity, Resistance surface, Glacier National Park, Hierarchical models, Cost-distance, *Ursus americanus*, oSCR

## Abstract

**Background:**

Improved understanding of wildlife population connectivity among protected area networks can support effective planning for the persistence of wildlife populations in the face of land use and climate change. Common approaches to estimating connectivity often rely on small samples of individuals without considering the spatial structure of populations, leading to limited understanding of how individual movement links to demography and population connectivity. Recently developed spatial capture-recapture (SCR) models provide a framework to formally connect inference about individual movement, connectivity, and population density, but few studies have applied this approach to empirical data to support connectivity planning.

**Methods:**

We used mark-recapture data collected from 924 genetic detections of 598 American black bears (*Ursus americanus*) in 2004 with SCR ecological distance models to simultaneously estimate density, landscape resistance to movement, and population connectivity in Glacier National Park northwest Montana, USA. We estimated density and movement parameters separately for males and females and used model estimates to calculate predicted density-weighted connectivity surfaces.

**Results:**

Model results indicated that landscape structure influences black bear density and space use in Glacier. The mean density estimate was 16.08 bears/100 km^2^ (95% CI 12.52–20.6) for females and 9.27 bears/100 km^2^ (95% CI 7.70–11.14) for males. Density increased with forest cover for both sexes. For male black bears, density decreased at higher grizzly bear (*Ursus arctos*) densities. Drainages, valley bottoms, and riparian vegetation decreased estimates of landscape resistance to movement for male and female bears. For males, forest cover also decreased estimated resistance to movement, but a transportation corridor bisecting the study area strongly increased resistance to movement presenting a barrier to connectivity.

**Conclusions:**

Density-weighed connectivity surfaces highlighted areas important for population connectivity that were distinct from areas with high potential connectivity. For black bears in Glacier and surrounding landscapes, consideration of both vegetation and valley topography could inform the placement of underpasses along the transportation corridor in areas characterized by both high population density and potential connectivity. Our study demonstrates that the SCR ecological distance model can provide biologically realistic, spatially explicit predictions to support movement connectivity planning across large landscapes.

**Supplementary Information:**

The online version contains supplementary material available at 10.1186/s40462-023-00445-7.

## Background

The loss and restriction of animal movement because of anthropogenic land use change increasingly challenges the conservation and management of terrestrial wildlife populations [1–3]. Land development and growing human population density surrounding protected areas has increased isolation of protected areas [4–6]. Isolation reduces the effective size of protected areas which can increase extinction risk for some large mammal populations [7–11]. These threats to the integrity and connectivity of protected area networks intensify in the context of rapid climate change as animals need to move to access bioclimatically suitable habitat [12–14]. Animal movement profoundly affects individual fitness and survival, the structure and size of populations, and ecosystem processes [15–19]. Thus, the conservation and restoration of ecological connectivity, including animal movement among protected area networks, has become a globally important strategy to support the resilience of wildlife populations [20–24].

Connectivity is the “the degree to which a landscape impedes or facilitates movement between resource patches” [25]. A functional perspective applies connectivity to animal movement and behavior, which can include how landscapes impede or facilitate within home-range movements, natal dispersal, and seasonal migration [26, 27]. Although multiple quantitative geospatial modeling approaches have been developed to identify areas that maximize functional connectivity, least-cost distance models underlie most quantitative corridor applications [28–31]. Cost-distance models use graph theory to discretize landscapes as a collection of cells connected by edges. Cell values represent the cost for an animal to move across the distance of the cell, and the least cost path (corridor) is calculated as the cumulative, shortest cost-weighted distance between two locations [28, 32]. Least cost-distance models thus assume that the path which minimizes cost-distance best represents animal movement.

All connectivity models regardless of the algorithm require cell specific landscape resistance values as inputs. Resistance values represent the ecological costs of movement such as energy expenditure or mortality risk associated with landscapes and are inversely proportional to connectivity values [33–35]. Species-specific resistance values are typically based on animal movement observations and have been directly estimated with analytical approaches (e.g. [36, 37]) or inferred from resource- or step-selection functions. Selection functions compare landscape features where animals or movement steps were observed (via GPS/VHF telemetry) to locations where movement was not observed, and resistance values are calculated from selection coefficients as an inverse function of selection probability [38–41]. Underlying selection approaches to inferring resistance is the assumption that selection is an accurate measure of an animal’s ability or willingness to move across a spatial unit. While movement data analyzed with a selection framework can provide more accurate information about landscape resistance compared to expert knowledge or animal presence/absence data [40, 42–44], this assumption may not hold true for all connectivity modeling applications [44, 45]. Overall, most connectivity modeling approaches do not consider how individual movement links with demography, despite evidence that demographic processes can influence individual movement and the connectivity of populations [46–49].

Spatial capture–recapture (SCR) provides a statistical framework to formally connect inference about individual movement to variable density of animals in wildlife populations [49–51]. SCR models are spatially explicit mark-recapture models that account for imperfect observation while estimating density from individual encounter data and provide inference about where and why a population is distributed in space. SCR models can be applied to data collected via non-invasive sampling techniques such as camera traps or DNA collected from hair traps, both of which are often used to sample wildlife populations across large landscapes [51–54]. SCR models classically formulate encounter probability as a function of Euclidean distance between trap locations and latent individual activity centers across the landscape which approximate individual home ranges during the study period [50, 51]. Recent advances in SCR modelling techniques have increased the potential for more robust inference about the relationship between individual space use and population density [49, 55]. For example, the ecological distance SCR model allows for simultaneous inference about population density and movement connectivity [37]. In the SCR ecological distance model, Euclidean distance in the encounter model is replaced with cost-distance, allowing for the analytical estimation of resistance values for any landscape feature and the calculation of population connectivity metrics informed by spatial variation in density [56–58].

The density-weighted SCR approach to estimating connectivity makes it possible to identify locations that have high potential connectivity *and* are likely used by a relatively greater number of individuals in the population of interest. This contrasts with approaches used in landscape genetics and most tracking studies which do not address the spatial distribution of individuals and wherein logistics can limit the ability to randomly sample individuals. Applying such approaches alone in connectivity planning could lead to prioritizing locations used by few individuals in accessible locations and therefore overlooking critical locations with higher population density. Estimating connectivity with a SCR approach thus offers potential advantages and trade-offs compared to a telemetry-based approach. In the SCR context, typically a large number of individuals are sampled and both demographic and movement parameters can be estimated, but the number of relocations per animal and the temporal resolution of relocations is lower. The SCR approach may thus be most appropriate for wide-ranging, elusive species such as large carnivores when deploying telemetry randomly is difficult or cost prohibitive, or population density is spatially structured. Despite the advances in SCR modeling, few studies have applied ecological distance SCR models to estimate landscape resistance to movement and most have been simulation-based [56, 58, 59]. Even fewer studies have applied SCR ecological distance models to a large, empirical dataset or have used this approach to support connectivity planning (for an exception, see [60]).

Here, we apply SCR ecological distance models to spatial capture–recapture data to estimate landscape resistance values and density-weighted population connectivity for American black bears (*Ursus americanus*) in Glacier National Park (henceforth Glacier) and surrounding landscapes. Glacier is at the center of the transboundary Crown of the Continent Ecosystem, one of the most intact ecosystems in North America, which spans over 7 million hectares of the Rocky Mountains in North America from Montana to British Columbia [61]. However, Glacier is partly situated in one of the fastest growing counties in Montana; 15% of all new homes built in Montana from 2000 to 2018 were built in Flathead County resulting in the conversion of 6% of open space to housing developments [62]. Visitation to Glacier has increased dramatically since 2000, reaching a peak of 3.3 million in 2017 and has hovered near 3 million in recent years [63]. In addition, the US highway 2 (hereafter US2) and Burlington Northern–Santa Fe railroad (BNSF) transportation corridor bisects contiguous protected areas in the region which could threaten the connectivity of wildlife. Traffic volume on US2 nearly doubled between 2001 and 2013, decreasing the frequency and duration of safe crossing periods for wildlife [64–66].

Previous studies in the region have estimated black bear density and abundance [67], seasonal sympatry with grizzly bears [68], and landscape genetics [69–71], but no study has assessed functional connectivity for black bears. Black bears are habitat generalists and although they are considered resilient to some urbanization, several studies have documented changes to black bear behavior, space use, and population dynamics in response to human development and disturbance [72–76]. Cushman et al. [29] used a landscape genetics approach to identify general areas for black bears to maintain connectivity from Yellowstone National Park to the Canadian border and found that a potential corridor area passed through the Great Bear Wilderness to Glacier across US2 and the BNSF railroad. Black bear mortalities due to train and vehicle strikes occur in the corridor, but effects of the US 2 and BNSF railroad transportation corridor on black bear population connectivity remain largely unknown [65].

Our objectives were to (1) estimate black bear population connectivity and understand how the transportation corridor may impact connectivity, (2) investigate sex-based differences in landscape resistance to movement, and (3) develop maps to inform landscape connectivity planning. We expected that the transportation corridor, paved roads, and high-relief terrain could impede bear movement whereas forest cover, riparian habitat, and drainage networks may facilitate bear movement as several studies have identified positive relationships between forest cover and riparian habitat and black bear space use [58, 77, 78]. We expected that due to differences in dispersal and movement behavior of reproductive females that female bears might avoid road crossings more than males, and that females might use dense forest more than male bears [78–80].We discuss how our study contributes new understanding of black bear spatial ecology in the region and we demonstrate how our results can be applied to support landscape connectivity planning for land managers in the Crown of the Continent Ecosystem.

## Methods

### Study area

**Fig. 1:**
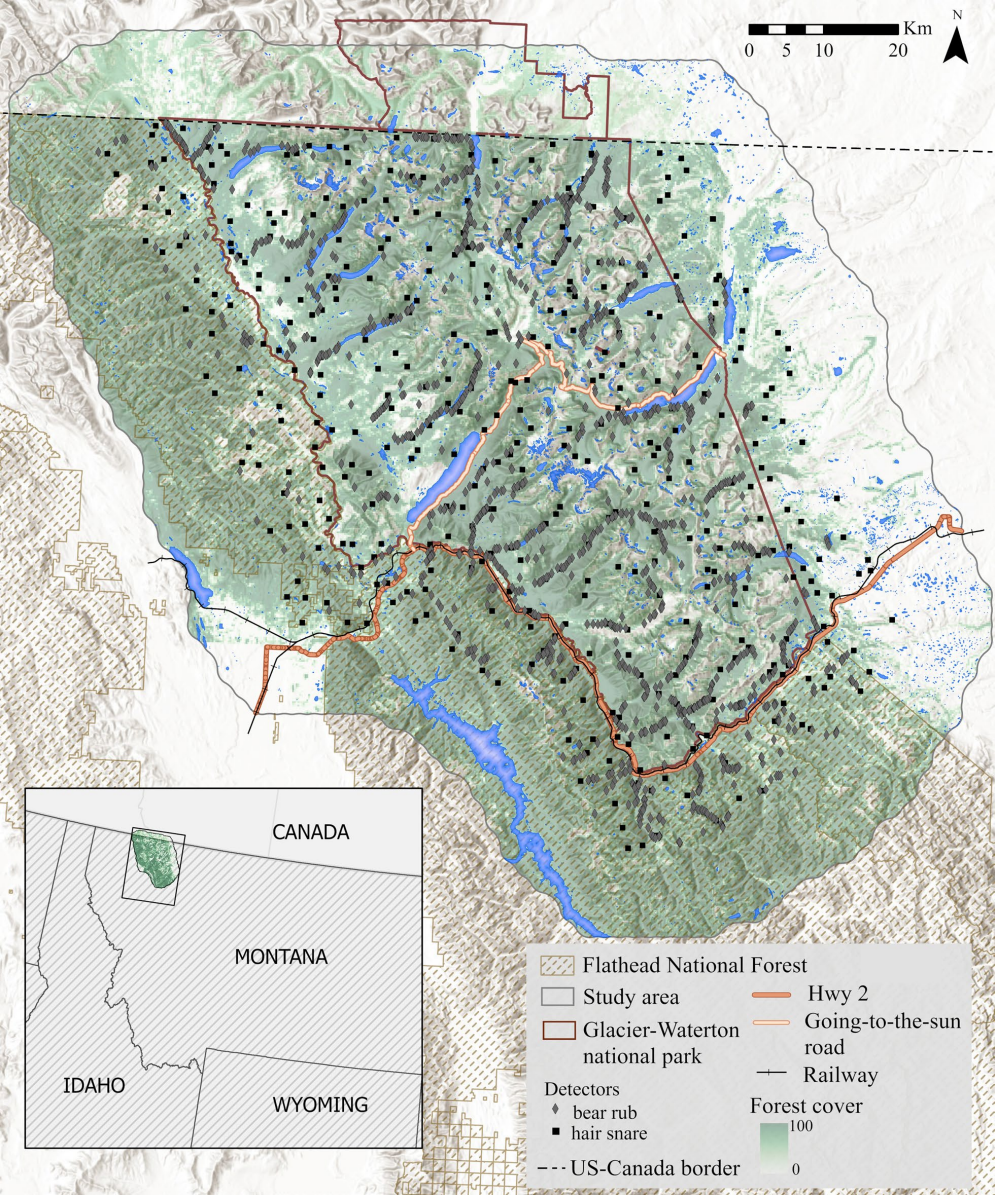
Map of study area including locations of bear rub (n = 1478*)* and hair snare (n = 438) detectors used in this black bear spatial capture-recapture study, 2004, Montana, USA

Our analyses centered on Glacier National Park (Glacier) and the northern Great Bear Wilderness in northwest Montana and covered an area of 10,936 km^2^ extending north into the southern Canadian Rockies and east to the Blackfeet Reservation (Fig. 1). Salish, Kootenai, Amskapi Piikuni (Blackfeet), and Sisika Indigenous peoples have shaped vegetation communities across the landscape through ecosystem maintenance and management practices that began thousands of years ago [81–83]. The landscape is dominated by the Rocky Mountains and runs along the continental divide with elevation ranging from 960 to 3190 m. West of the divide, coniferous forests primarily comprised of Engelmann spruce (*Picea engelmannii*) and subalpine fir (*Abies lasiocarpa*) dominate due to greater precipitation compared to the drier climate east of the continental divide which supports more aspen (*Populus tremuloides*) and shortgrass prairie at lower elevations. Most of the study area is protected federal land that supports all pre-settlement native North American large mammal species except buffalo (*Bison bison*), including sympatric populations of grizzly bears (*Ursus arctos*) and American black bears (*Ursus americanus*). The region also includes a mosaic of private lands, tribal lands, and national forest used for timber harvest and recreation. Mixed-use, developed lands, and denser networks of roads are primarily outside of Glacier in nearby river valleys and along the Middle Fork Flathead River-US2-BNSF railroad corridor that separates Glacier to the north from the federal wilderness area to the south (Fig. 1). The southwest corner of the study area includes the small towns of Columbia Falls and Whitefish. Inside Glacier, the Going-to-the-Sun Road runs nearly 50 miles east–west across the park, crossing the continental divide at Logan Pass and only a few other short roads provide access up to ~ 10 miles into the park.

### ***Genetic ****capture–recapture**** data***

From 3 June to 11 October 2004, the U.S. Geological Survey led a project that simultaneously implemented two noninvasive sampling methods, hair snares and bear rubs, to collect bear hair samples across the study area as a part of a larger project described in Kendall et al. 2009 (Fig. 1; [52]). Hair snares (n = 438) consisted of a strand of barbed wire stretched between 3 and 6 trees with a liquid scent lure of cattle blood and decomposed fish in the center [52]. Researchers deployed one hair snare per 7 × 7-km grid across the landscape for approximately 14 days, after which all hair samples were collected, and the trap was moved > 1 km in the same grid [52]. Researchers opportunistically sampled bear rubs (n = 1478) by attaching several strands of barbed wire to vertical objects (i.e., a tree or post) with smoothed bark, scratches, hair, or other sign that bears rubbed on them and removed hair every ~ 14–20 days [52]. Species and individual identity were assigned to each hair sample through genetic analyses of six microsatellite loci, and sex was assigned using the amelogenin marker (for analyses details, see [67]). Subsampling black bear hairs reduced genotyping costs, as detailed in [67], and resulted in 1019 samples used for identifying black bear individuals [84].

### ***Ecological distance spatial ****capture–recapture**** models***

To estimate density and connectivity of black bears in Glacier, we fit SCR models to the individual bear encounter histories using maximum likelihood estimation in the package ‘*oSCR’* (version 0.42.0 [85];) implemented in R (version 4.0.5 [86];). SCR analysis combines a spatially explicit encounter model conditional on a spatially explicit point process model of latent individual activity centers ${({\varvec{s}}}_{{\varvec{i}}}$**)** distributed across a discrete space (state space, ${\varvec{S}}$) containing all capturable individuals in the population of interest [51]. Every individual in the target population has an activity center, which is treated as the centroid of an individual animal’s home range during the sampling period [37]. The activity center is a latent variable estimated by the model. In our model, $i$ individual detections (or not) at $j =1914$ hair snares and bear rubs with coordinates ${{\varvec{x}}}_{{\varvec{j}}}=\{{x1}_{j}, {x2}_{j}$} across $k=6$ sampling occasions are assumed to be Bernoulli random variables such that ${y}_{ijk}\sim {\text{Bernoulli}}(p{}_{ij})$. Density is estimated as the number of estimated individual activity centers divided by the area of the state space and can be modeled as a function of spatial covariates measured at the resolution of ${\varvec{S}}$. Encounter probability is linked to the spatial point process by letting the probability of detecting an individual at a particular detector $(p{}_{ij})$ vary as function of distance from that individual’s activity center ${{\varvec{s}}}_{i}$ to the detector location ${{\varvec{x}}}_{j}$. Standard SCR encounter probability models rely on Euclidean distance as is the case with the standard half-normal model in which:

1 $$p_{ij} = p_{0} \times \exp \left[ {{\raise0.7ex\hbox{${{-\text{dist}}_{euc} \left( {x_{j} ,s_{i} } \right)^{2} }$} \!\mathord{\left/ {\vphantom {{{\text{dist}}_{euc} \left( {x_{j} ,s_{i} } \right)^{2} } {2\sigma^{2} }}}\right.\kern-0pt} \!\lower0.7ex\hbox{${2\sigma^{2} }$}}} \right]$$

where ${p}_{0}$ is the baseline encounter probability and $\sigma$ is the spatial scale parameter that determines the rate of decline in detection probability as the Euclidean distance between activity center ${{\varvec{s}}}_{{\varvec{i}}}$ and detector ${{\varvec{x}}}_{{\varvec{j}}}$ increases. Euclidean distance-based models assume that animal space use is symmetric and circular regardless of landscape structure and is stationary on the activity center ${\varvec{s}}$. Here, we utilize the recently developed ecological distance SCR model [37, 56] in which Euclidean distance in the encounter probability model (Eq. 1) is replaced with the length of the least cost path or ‘ecological’ distance between two points on the landscape $\left({\nu }_{0}\right.$ and ${\nu }_{T})$:

2 $${dist}_{lcp}\left({\nu }_{0},{\nu }_{T}\right)=\underset{{L}_{1,\dots ,; {L}_{W}}}{{\text{min}}} \sum_{a=0}^{T}\mathit{cost}\left({\nu }_{a},{\nu }_{a+1 }\right)\times {\mathrm{ dist}}_{euc }\left({\nu }_{a},{\nu }_{a+1}\right)$$

where for all possible paths (w = 1, …; *W* paths of length $L$) consisting of $T$ segments connecting adjacent cells (${\nu }_{a},{\nu }_{a+1 })$, the least cost path between ${{\varvec{\nu}}}_{0}$ and ${\nu }_{T}$ is the minimum of the length of the path (number of segments) multiplied by the associated cost of the covariate surface. Following Royle et al. [37], the $cost\left({\nu }_{a},{\nu }_{a+1}\right)$ is defined as a log-linear function of the average cell-specific values of covariate$z$:

3 $$cost\left({\nu }_{a},{\nu }_{a+1 }\right)=\frac{ exp ({\delta }_{z}\left({{\varvec{\nu}}}_{{\varvec{a}}}\right)) + exp ({\delta }_{z}\left({{\varvec{\nu}}}_{{\varvec{a}}+1}\right))}{2}$$

The inclusion of the cost equation, replacing Euclidean distance with ecological distance, allows the estimation of one or more resistance parameters (δ) that characterize the cell-specific cost of movement between any cells for a given raster covariate surface, and so allows for asymmetric detection and kernel shape in the encounter model that is directly related to space use by individuals [37]. Thus, we can calculate the expected probability of use ($$g)$$ for any cell $${s}_{u}\in {\varvec{S}}$$, by an individual with an activity center in cell $${s}_{i}$$ by evaluating Eq. 1 at the maximum likelihood estimates of δ and $$\sigma$$ and setting $${p}_{0}=1:$$

4 $$Pr(g[{s}_{u},{s}_{i}])={\text{exp}}\left(-\frac{1}{{2\sigma }^{2}} * {{dist}^{2}}_{ecol}[{s}_{u},{s}_{i}]\right)$$

This modified kernel accounts for resistance to movement between cells and therefore provides model estimates of the relationship between individual movement within the home range and landscape structure; a direct measure of local landscape connectivity during the time of sampling [37, 56]. In addition, SCR models based solely on Euclidean distance often mis-specify animal space use, which can negatively bias density (and abundance) estimates due to the presence of unmodeled heterogeneity in detection probability that is related to the assumption of symmetric, stationary home ranges [37, 87].

In SCR analyses, the state space must be large enough to include all potential animals with non-negligible probabilities of detection based on the trap locations [51]. We buffered the outermost traps in our study area by 12 km, 3 × the male sigma estimate from a previous study to set the extent of the state space [88, 89]. We excluded lakes > 1 ha, mountain peaks with persistent ice/snow cover, and barren rock/cliff faces as locations unavailable for activity centers. We used a spatial resolution of 2 km based on the recommendation that the resolution should be less than the expected estimate of sigma [85]. Ecological distance SCR models implemented in *oSCR* also require a cost space to estimate δ containing covariates hypothesized to affect animal movement. Following guidance in Sutherland et al. [60], we set the resolution of the cost space to 1/16 of the resolution of the state space (0.25 km). For efficiency given the large dataset and because the probability of detection approaches zero as the distance from the activity center increases, we fit all models such that only locations within a plausible distance (‘trimS’) of capture locations were included as possible locations for the activity center of an individual. We used distances of 36 km for females and 76 km for males and tested the sensitivity of parameter estimates to the maximum distance to ensure that estimates were stable.

We fit separate models for male and female black bears because females have smaller home ranges [67, 90]. Additionally, few studies have evaluated sex-based differences in movement costs although male and female bears likely use space differently [33, 91]. For example, female bears may avoid use of developed areas more than male bears [92, 93]. For each sex, we tested for a rubbing behavior effect on detection at bear rubs. Bear rubbing behavior at bear rubs likely signals a bear’s presence chemically to other individuals and has primarily been identified in brown bears [94–96] but rarely studied in American black bears. In our models, the inclusion of a trap specific behavioral response indexes how the use of a specific rub may change the probability that a bear (the same individual or another) will rub there in the future [97].

### Environmental data

We developed environmental descriptors and candidate models based on a priori knowledge of American black bear ecology as several previous studies have estimated black bear densities at sites across North America [54, 76, 93]. To model spatial variation in black bear detection probabilities and densities, we considered variables indexing variation in human land-use, vegetation structure including impacts of recent fires, vegetative food availability, terrain, and land management regimes across the study area (Additional file 1). We also considered a covariate indexing grizzly bear space use across the study area because competitive interactions between sympatric populations of grizzly and black bears may lead either species to avoid the other in systems where they rely on similar food sources [68, 90]. We include detailed descriptions of all covariates in Additional file 1 and focus here on covariates developed to index potential variation in connectivity for black bears across the study area (Table 2).

We explicitly sought to test for an effect of the US2 and BNSF railroad transportation corridor on black bear movement connectivity, so we extracted all cells that intersected either the highway, the railroad, or both (Fig. 1). We also extracted all cells intersecting the Going-to-the-Sun Road in Glacier hypothesizing that it may increase movement cost for bears due to high tourism traffic during daylight hours and to test for different effects of a low-speed road in the protected area vs a river-highway-railroad corridor with high-speed traffic in unprotected land. We calculated paved road density across the study area (km/2 km^2^) because black bears can avoid areas of high road density [73, 98, 99].

We investigated several landscape features that we hypothesized might facilitate connectivity because this information can be used to support landscape planning, specifically: vegetation cover, drainage networks, and topography [58, 77, 78]. We calculated percent forest cover across the study area and extracted categorical riparian vegetation and deciduous forest map layers from LANDFIRE data [100]. We extracted the hydrology network from the National Hydrography Dataset [101] for the study area, hypothesizing that drainages networks and valley bottoms may be used as transit corridors by bears [102]. We also calculated the mean and standard deviation of total terrain curvature derived from the National Elevation Dataset Digital Elevation Model (DEM) (30 m; [103]) to account for variation in terrain complexity and differentiate continuous slopes from broken slopes because we expected that high-relief terrain could impede bear movement and that flatter terrain may facilitate movement [97, 104]. For all linear landscape features, we created both a binary covariate representing presence or absence of the feature in each cell and a continuous, ‘distance to’ representation of the feature by calculating the cumulative surface distance from the feature to all other cells in the cost space (Table 1). Instead of letting the distance accumulate unbounded, we set the maximum distance at a threshold of 76 km, three times the longest-distance movements made by bears in the dataset. We processed all spatial data using R [86] and the packages *dplyr* [105] and *raster* [106]. We tested for multicollinearity in the covariates and did not include covariates in the same model if pairwise correlation coefficients were >|0.65|.

**Table 1:** List of covariates hypothesized to influence movement cost for black bears and tested in SCR ecological distance models in Glacier National Park, Montana, USA, 2004

SCR resistance parameter (**δ**)	Covariate	Expected effect on resistance to movement	Evidence
**δ** (resistance parameter, i.e., movement cost)	Paved road density	Increase	[73, 98]
	Distance to paved roads	Decrease	″
	BNSF railway	Increase	[66, 70, 71, 107]
	US2 presence/absence	Increase	″
	Distance to US2/BNSF railway	Decrease	″
	Transport corridor: linear combination of US2 and railway	Increase	″
	Going-to-the-sun Road	Increase	″
	Terrain curvature (SD)	Increase	[68, 70]
	Terrain curvature (Mean)	Increase	″
	Aspen/deciduous forest presence/absence	Decrease	[102]
	Riparian vegetation presence/absence	Decrease	″
	Distance to drainage	Increase	[102, 108]
	Drainage present	Decrease	″
	Forest cover (all)	Decrease	[58, 67, 78]

### Model selection

We used a multi-stage modeling approach to build ecologically relevant candidate models as testing all possible combinations of detection, density, and connectivity covariates would have resulted in an unreasonably large number of possible model combinations and because SCR ecological distance models require long runtimes [109, 110]. We first fit density sub-models for each covariate that we hypothesized to influence density with a plausible model for variation in *p0* that included trap-specific effects on detection (i.e., trap effort, trap type, detection date, forest cover) strongly supported in similar genetic capture–recapture studies [67, 97]. We ranked the univariate density sub-models using Akaike’s information criterion (AIC) [111]. At this stage, we eliminated uninformative spatial covariates resulting in heterogenous density models with similar log-likelihood values and equal or lesser Akaike weights than that of the homogenous (null) density model and covariates with responses indicating they did not correctly index the biological mechanism related to our hypotheses [112, 113]. If any two remaining covariates were colinear, we proceeded with the variable that was more supported based on AIC.

We next paired the most supported sub-model for density (forest cover) identified in the density covariate reduction step with all sub-models for detection covariates following an all plausible combinations covariate reduction strategy identified by Bromaghin et al. [114]. We considered only sub-models for detection with both high weight and high likelihood from this set as plausible. We then used these detection models to test all combinations of plausible sub-models for density and detection as candidate models (Additional file 2: Tables S1 and S2) [114]. We used coefficients generated during the univariate modeling stage as starting values to support model convergence [85]. We identified the most parsimonious combination of density and detection model structures using AIC and then used this model structure in univariate connectivity models to evaluate covariates hypothesized to influence resistance. We used the approach described above to eliminate uninformative cost covariates from further consideration by comparing log-likelihood values and Akaike weights with those of the Euclidean distance (null cost) model. In addition, because we tested different functional forms of cost covariates (e.g., US2 presence/absence vs. distance to US2), this approach allowed us to advance only the most supported functional form of each covariate to the final candidate model set [112]. Finally, we constructed full ecological distance models with all combinations of remaining informative cost covariates (Additional file 2: Tables S3 and S4). We based our inference on the best AIC ranked full ecological distance model for each sex and used the coefficient estimates from these models to calculate realized (predicted) density and population connectivity surfaces.

### Calculating landscape population connectivity

The estimation of covariate-specific resistance parameters provides a measure of local connectivity by allowing the probability of space use (i.e., movement) within an individual’s home range to be influenced by landscape variation as shown in Eqs. (3 and 4). Following the approach outlined in Sutherland et al. [56] and Morin et al. [58] we extend Eq. 4 to estimate the probability of use for all cells in the landscape and derive a direct measure of landscape connectivity for any number of activity centers (${{\varvec{s}}}_{{\varvec{i}}}$) [56, 58]. We used the maximum likelihood estimates of the resistance parameters (${\delta }_{1},\dots ;{\delta }_{x}$) and $\sigma$ the kernel scaling parameter, to compute cell specific connectivity across the landscape (i.e., the potential connectivity ($PC{(s}_{u})$) surface; [56, 57]). Potential connectivity is the expected number of individuals that would use each cell when each cell in ${\varvec{S}}$ contains a single activity center and represents how connected each cell is to all other cells in the landscape:

$${PC(s}_{u})= \sum_{{S}_{i}\in {\varvec{S}}\boldsymbol{ }}{\text{exp}}\left(-\frac{1}{{2\sigma }^{2}} * {{dist}^{2}}_{ecol}{[s}_{u, }{s}_{i}]\right)$$

In addition, we estimated density-weighted connectivity (DWC) called “realized connectivity” in Sutherland et al. [56] and “DWC” in Morin et al. [58], which is a measure of landscape connectivity that combines realized density and potential connectivity by weighting the cell-specific potential connectivity value by the model estimated density of each cell:

$${DWC(s}_{u})= \sum_{{S}_{i}\in {\varvec{S}}\boldsymbol{ }}{\text{exp}}\left(-\frac{1}{{2\sigma }^{2}} * {{dist}^{2}}_{ecol}{[s}_{u, }{s}_{i}]\right) *D({s}_{i})$$

The DWC surface thus describes the ability of individuals to move through a landscape with respect to the spatial distribution of individuals across the landscape, providing a direct estimate of landscape population connectivity. We used the DWC surfaces to identify locations predicted to best support black bear population connectivity across the transportation corridor based on DWC values. We summed the male and female surfaces to estimate a total DWC value for each pixel within 1 km of U2 and then identified high-use zones predicted to be used by the greatest number of bears (highest DWC values) to support mitigation and connectivity planning efforts [84].

We found several data handling and model fitting nuances were required to apply the ecological distance SCR model to a large empirical dataset across a large study area (> 10,000 km^2^) at a relatively fine grain size (250 m). We include example code  for fitting SCR ecological distance models and calculating density-weighted connectivity  surfaces in Additional file 3.

## Results

Genotyping hair samples resulted in 924 detections of 598 individual bears (295 males and 303 females) during the study period (Table 2). We report the ratio of simple (i.e., same trap) recaptures to spatial (i.e., > 1 trap) recaptures, the number of unique individuals with spatial recaptures, and the distances moved between spatial recaptures because these data characteristics and sample sizes of each have the potential to affect the accuracy and precision of SCR parameter estimates [115]. Of the 598 black bears detected, 194 were recaptured at least once and 99% of these recaptures were spatial, resulting in 911 total spatial detections.

**Table 2:** Summary of black bear detection data collected from June-October 2004 used in spatial capture-recapture analyses

Detected		Individuals				Detections		
		Recaptures^a^	Spatial recaptures^b^	Spatial: all recaptures^c^	Total	Spatial	Distance IQR (km)^d^	MMDM (km)^e^
Females	303	80	79	0.98	430	425	0.89–4.32	3.2
Males	295	114	114	1.00	494	486	2.6–8.6	7.8
Total	598	194	193	0.99	924	911		

^a^Multiple detections of the same individual

^b^Multiple detections of the same individual at trap locations that differ from the previous detection

^c^The ratio of individuals with spatial recaptures to the total number of recaptured individuals

^d^The interquartile range (IQR) of distances moved across individuals in kilometers

^e^The mean maximum distance moved (MMDM) across individuals in kilometers

**Fig. 2:**
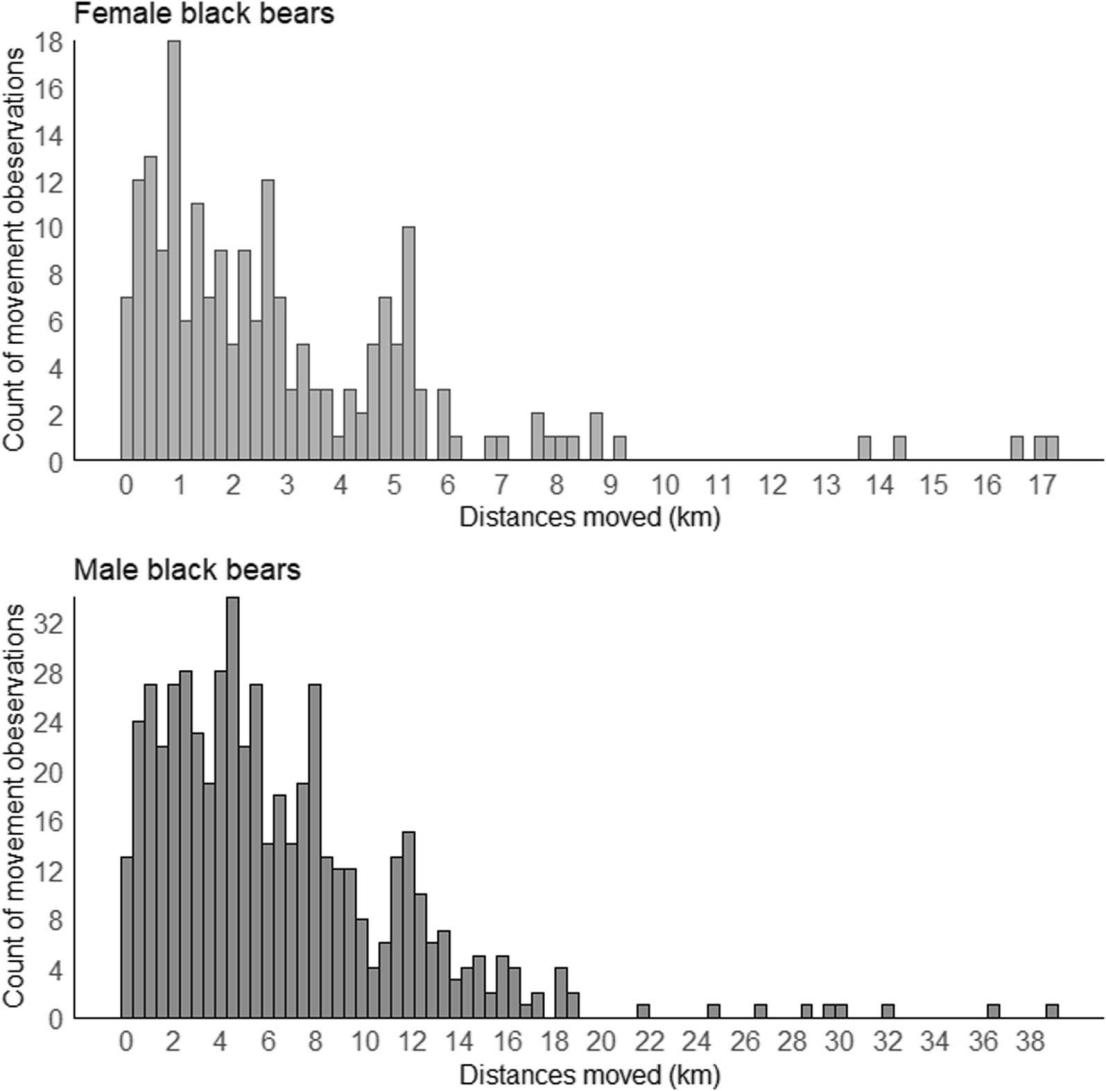
Histograms of movement observations and the distances moved by 79 female and 114 male black bears that were spatially recaptured in 2004, Montana, USA

The 79 females with spatial recaptures were captured at an average of 2.5 traps each (range = 2–7 unique traps), resulting in 199 unique observations of movement across distances ranging from 50 m to 17.2 km (Fig. 2). The 114 males that were spatially recaptured were recaptured at an average of 2.7 traps (range = 2–27) resulting in 533 unique movements across distances ranging from 80 m to 38.9 km (Fig. 2).

### Density and detection

Heterogenous density models were supported over the homogenous density model (Additional file 2: Tables S1, S2) for both sexes. For female black bears, the most supported model included a positive effect of percent forest cover on density, and this was the only informative covariate (Additional file 2: Tables S1, S2). The mean female black bear density estimate for the study area was 16.08 bears/100 km^2^ (95% CI 12.52–20.6) and predicted (realized) density estimates ranged from 4.4 to 42.3 bears/100 km^2^ across the study area, whereas the mean male black bear density estimate was 9.27 bears/100 km^2^ (95% CI 7.70–11.14) and predicted density estimates across the study area ranged from 1.07 to 21.2 bears/100 km^2^ (Fig. 2). Male black bear density was also positively related to percent forest cover, but the most supported model also included a negative effect of estimated grizzly bear densities in 2004 which were higher inside Glacier (Table 3). As a result, areas with the highest realized density estimates for males were in forests along the Glacier boundary whereas female densities were highest in contiguous forests both inside the park and out (Fig. 2).

**Table 3:** Best AIC-ranked ecological distance SCR model results for female and male black bears in Glacier National Park, Montana, USA, including untransformed maximum likelihood estimates (MLE), associated standard errors (SE), and 95% confidence intervals (CI)

Parameter and coefficient description		MLE	SE	95% CI	MLE	SE	95% CI
		Females			Males		
***p0*** (baseline detection probability)	Intercept (bear rub)	− 6.20	0.30	− 6.8 to − 5.61	− 7.77	0.29	− 8.37 to 7.17
	*β* rub behavior	0.99	0.10	0.79–1.20	1.35	0.09	1.16–1.54
	*β* bear rub effort	− 0.04	0.09	− 0.03 to 0.33	0.16	0.07	0.02 to 0.29
	*β* hair snare	0.73	0.37	0.01–1.46	− 0.05	0.30	− 0.64 to 0.53
	*β* hair snare effort	2.47	0.34	1.79–3.14	3.63	0.31	3.02–4.24
	*β* forest cover	0.15	0.08	0.01–0.30	0.09	0.07	− 0.03 to 0.23
	*β* Julian day	0.25	0.09	0.07–0.42	5.05	0.49	4.09–6.01
	*β* std. of terrain curvature	− 0.09	0.06	− 0.22 to 0.02	− 0.22	0.06	− 0.33 to − 0.10
	*β* Julian day quadratic effect				− 1.95	0.21	− 2.36 to − 1.53
**σ** (spatial scaling)	Intercept	0.55	0.09	0.36–0.74	1.04	0.21	0.63–1.45
**δ** (resistance or cost of movement)	*β* drainages binary	− 0.53	0.15	− 0.83 to − 0.23			
	*β* forest cover				− 0.85	0.29	− 1.41 to − 0.29
	*β* distance to drainages				1.50	0.45	0.62–2.39
	*β* transportation corridor				2.20	0.30	1.61–2.79
**Density** (bears per 4km^2^ pixel)	Intercept (D_0_)	− 0.44	0.13	− 0.69 to − 0.19	− 0.99	0.09	− 1.81 to − 0.81
	*β* forest cover	0.22	0.11	0.00–0.44	0.04	0.19	− 0.33 to 0.42
	*β* grizzly density				− 0.18	0.09	− 0.35 to − 0.01

For both sexes, the best supported models included variation in detection probability with trap type, trapping effort, rubbing behavior, forest cover, terrain curvature, and Julian day (Table 3). Detection probabilities were higher at baited hair snares than at bear rubs for both sexes, but there were 1438 rubs and only 438 hair snares. We found a strong positive effect of rubbing behavior on detection probability at bear rubs (*β*_females_ = 0.99 [0.79–1.20], *β*_males_ = 1.35 [1.16–1.54]; Table 3). The predicted probability of detection at a bear rub used more than once was 150% greater on average for females and 100% greater on average for males compared to rubs used only once by a single bear during the study period (Additional file 2: Figure S1). Detection probability was generally highest in mid-late summer (July–August) and for male bears, change in detection probability over time was best described by a quadratic effect (Table 3, Additional file 2: Fig. S1). The top models for both sexes also included a positive effect of forest cover and a negative effect of increasing terrain curvature at a trap on detection probability though these effects were weaker relative to other supported covariates (Table 3). Comprehensive model selection results for both males and females are in Additional file 2: Tables S1–S5.

### Space use and landscape resistance to movement

**Fig. 3:**
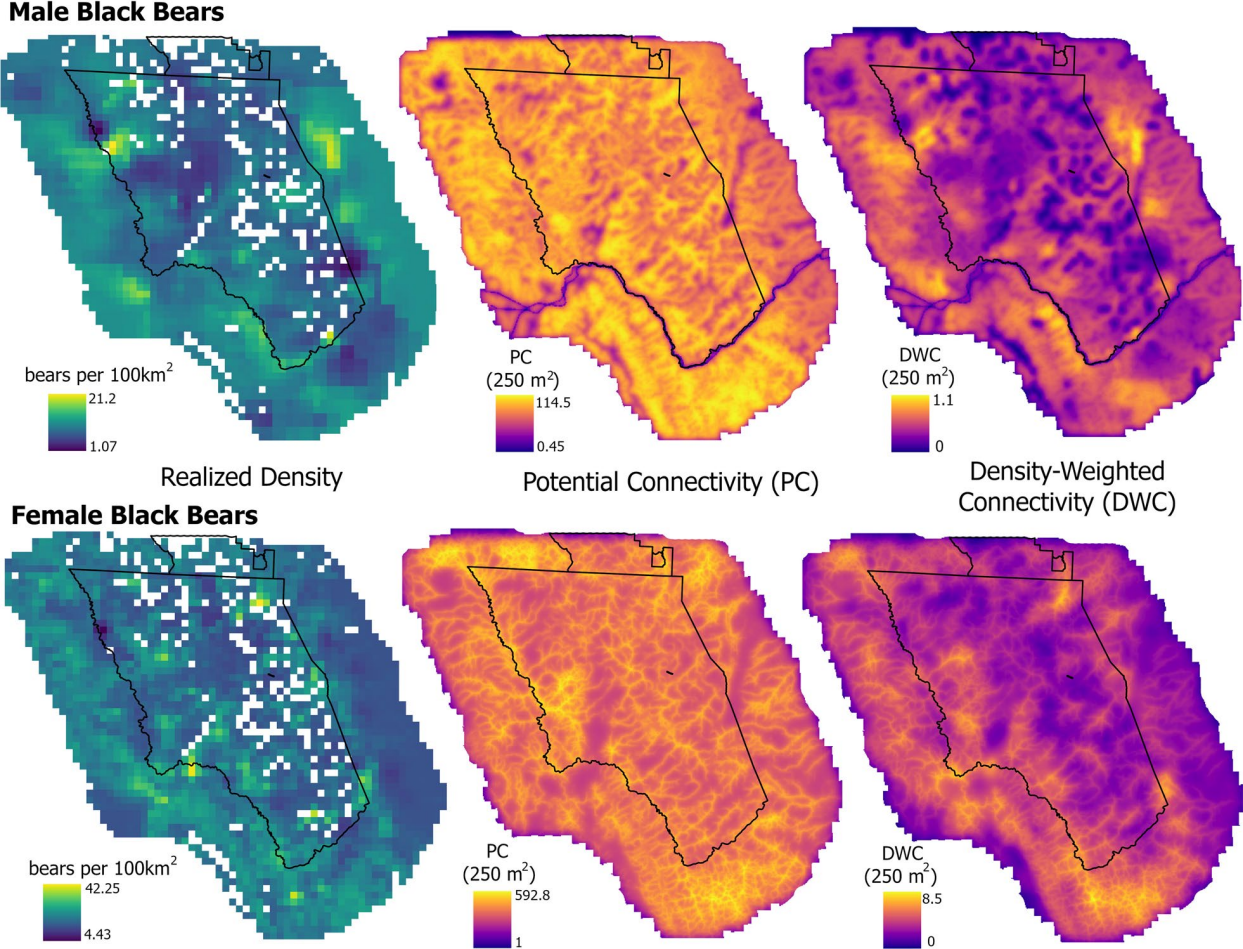
Maps displaying *realized* density, potential connectivity, and density-weighted connectivity across the study area for male and female black bears in 2004 in Montana, USA [84]. White areas were excluded as possible activity centers due to the presence of large lakes, rocks, or glaciers

For females, the best supported ecological distance model estimate for *σ* was a mean of 1.7 km (95% CI 1.43–2.1 km) whereas the average *σ* estimate for males was 2.8 km (95% CI 1.8–4.3 km). Model selection results indicated differences in how the landscape influenced the cost of movement (δ) for male and female bears. Both drainages and riparian vegetation cover were associated with decreased resistance to movement for female bears and these were the only models more informative than the null, Euclidean distance, movement model. Riparian cover and presence of drainages were spatially correlated so we used the most supported drainages model (Akaike weight = 0.68, Additional file 2: Table S4) to predict landscape population connectivity for female bears. Resistance to movement decreased in cells where streams and drainages were present (*β*_drainages binary_ = − 0.53 [− 0.83 to  − 0.23]; Table 3). For male black bears, the most supported ecological distance model (Akaike weight = 0.33, Additional file 2: Table S3) included variation in landscape resistance to movement with percent forest cover, distance to streams and drainages, and the transportation corridor (Table 3). For males, two other resistance models were within 2 ∆AIC of the most supported model (Additional file 2: Table S3). All three models contained effects of the transportation corridor and distance to drainages but differed with respect to vegetation effects: forest cover (top-ranked and used here for inference, riparian cover (rank 2), or no vegetation effect (rank 3). Resistance to movement for males increased in all cells that intersected US2, the BNSF railroad, or both (*β*_transportation corridor_ = 2.20 [1.61–2.79]; Table 3). Resistance also increased with increasing distance from drainages (*β*_distance to drainages_ = 1.50 [0.62–2.39]); and decreased with increasing percent forest cover for male black bears (*β*_forest cover_ =  − 0.85 [− 1.41 to  − 0.29]; Table 3, Fig. 3).

### Landscape population connectivity

Female black bear potential connectivity was highest where many drainages and valley bottoms were clustered in space, specifically in East Kootenay, British Columbia in the northwest corner of the study area, inside Glacier south of Logging Lake and along the North Fork Flathead River following the western boundary of Glacier, and in the Great Bear Wilderness south of Glacier (Fig. 3). The density-weighted connectivity (DWC) surface suggests the greatest realized connectivity for females occurs in the Great Bear Wilderness south of Glacier and surrounding West Glacier where there is a high density of streams and valley bottoms as well as a higher density of bears associated with more contiguous forest cover compared to the northwest portion of the study area (Fig. 3). Potential connectivity for male bears was lowest in cells intersecting both US2 and the railway followed by cells intersecting either feature, suggesting that the transportation corridor significantly increases resistance to movement for male black bears (Table 3, Fig. 3). Elsewhere on the landscape, the potential connectivity surface suggests little variation and generally high connectivity in all cells near drainages and high forest cover, particularly in the Great Bear Wilderness in the southernmost part of the study region.

**Fig. 4:**
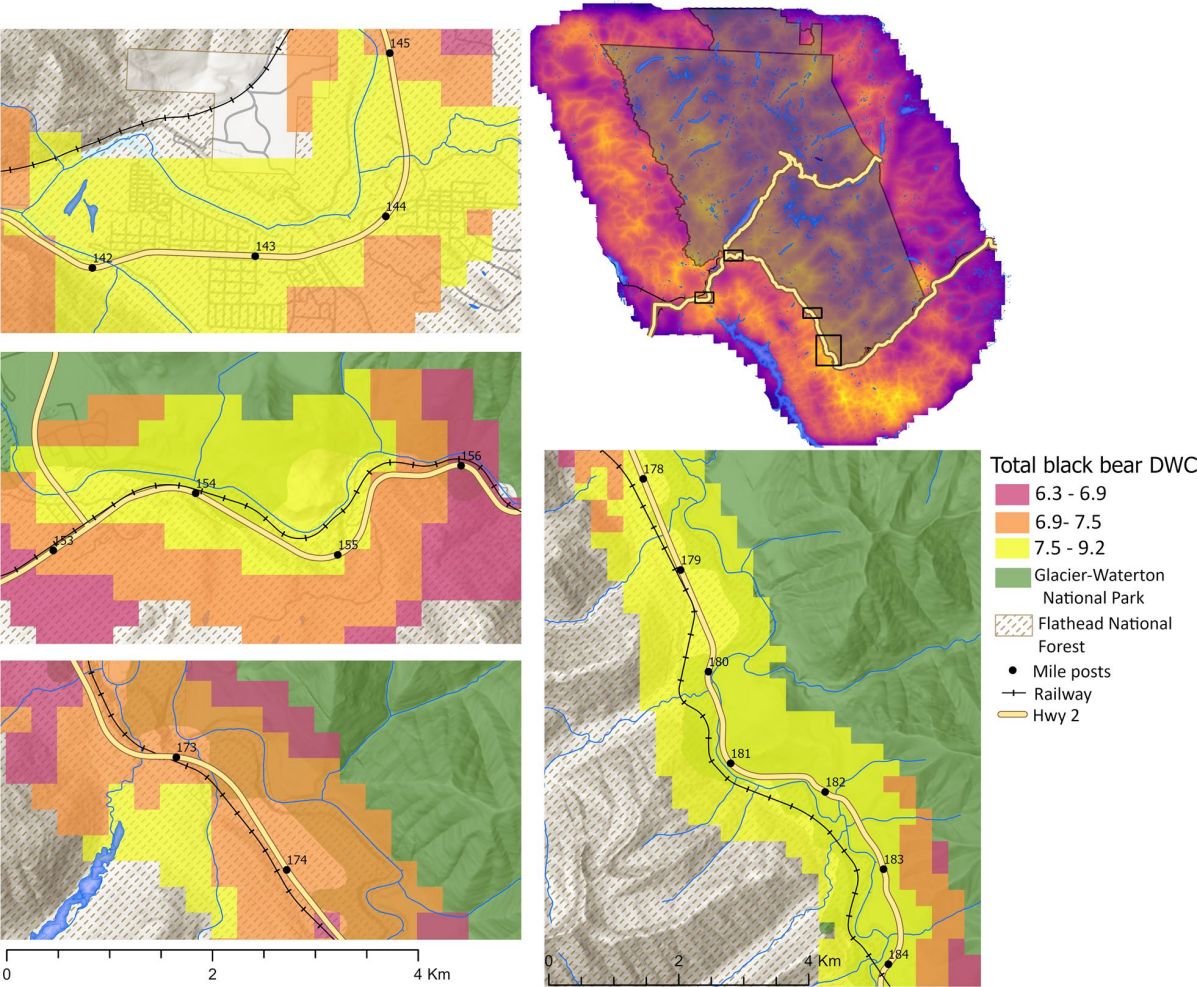
Predicted high use crossing zones for the US2–BNSF transportation corridor based on the highest total density-weighted connectivity (DWC) values for male and female American black bears in 2004 in Glacier National Park, Montana, USA. Inset maps are arranged from west to east and have black numbers representing mile markers

In contrast, the male DWC surface shows realized connectivity is highest in the northwestern portion of the study region in the Flathead National Forest and west of Bowman Lake inside Glacier, in the northeast part of the study region outside of Glacier along lower St. Mary Lake, and directly southwest of US2 in the Great Bear Wilderness (Fig. 3). Predicted total DWC across the transportation corridor was highest from mile posts 178–185 in valleys along Essex Creek, Sheep Creek, and Java Creek from the south as well as Ole Creek from the north (Fig. 4). Predicted total DWC values were also high from mile posts 153- 156 near West Glacier, between mile post 173 and 174 north of Pinnacle along Tunnel and Pinnacle Creek, and from mile posts 142–145 along the South Fork Flathead River west of Hungry Horse and north of Martin city along Abbot Creek (Fig. 4).

## Discussion

Both US2 and the BNSF railroad were significant barriers to connectivity for male black bears, and the effect was greatest where US2 and the railway occur in proximity (< 250 m apart); a finding not previously reported for the study area. In particular, the railway and highway had an additive effect resulting in an 800% predicted increase in resistance where both features occur (e.g., < 250 m apart) compared to cells where either feature occurs independently. However, we also found that drainages and valley bottoms facilitated connectivity for both female and male black bears in the Crown of the Continent Ecosystem. Simultaneously estimating density and movement connectivity revealed that not all locations with high potential connectivity support movement for many animals because the ecological variables driving spatial variation in density differed from those influencing movement probability. We demonstrate how our density-weighted connectivity estimates can inform mitigation planning for US2 and the BNSF railroad by identifying high use zones that may facilitate connectivity across these barriers for the greatest number of bears.

Our finding that US2 was a barrier to connectivity aligns with landscape genetics and tracking studies that found highways can increase resistance to movement and gene flow in black bear populations across the Rocky Mountains [69–71, 99]. Other studies report that black bears perceive risk associated with crossing roads, adjust their activity patterns to avoid periods of high road activity, and that bears living in rural areas may exhibit stronger avoidance of roads than bears living in regions with a higher proportion of developed land [72, 116, 117]. Few studies have examined the effects of railways on wildlife beyond documenting mortalities [118]. But evidence primarily from studies of brown bears in Europe and Canada suggests that train strike mortalities can significantly impact populations of wide-ranging wildlife where railways traverse protected areas [119–121].These railways can be dangerous ecological traps for bears because they are often associated with attractants including spilled grain from train cars and train-killed ungulate carcasses, resulting in increased mortality rates as a result of seeking such resources [121–124].

However, fewer studies have considered potential synergistic impacts where highways and railways occur in proximity [71, 123]. In a study of grizzly bear movements in Glacier, Waller and Servheen [125] found that grizzly bears avoided high-traffic periods on US2, making them more likely to attempt crossing the BNSF railroad at night when railroad traffic volume is high, resulting in higher grizzly bear mortality on the railroad than on the highway [123]. A study in a similar system further west found that among 16 black bears tracked for 1–2 years between 2005 and 2010 none crossed US2, the BNSF railroad, and the river, and two males crossed only US2 [71]. Additionally, we did not find evidence that the Going-to-the-Sun Road impacted black bear movement connectivity, although traffic volumes on US2 and Going-to-the-Sun Road are similar. Overall, these results suggest that US2, and the combination of a highway and railway in proximity has a greater impact on black bear movement connectivity than a single road. The Middle Fork Flathead River which parallels US2 for several miles and may only be crossable for bears in limited locations, especially during high water, may also increase resistance to black bear movement in the Middle Fork Flathead River-US2-BNSF railroad transportation corridor.

The transportation corridor may increase mortality risk in addition to the resistance to movement we measured. Resistance to movement and mortality risk are not equivalent and may influence resistance and connectivity differently [125]. Thus, additional research on the effects of the transportation corridor on black bear movements and mortality could be useful to evaluate if there are similar synergistic impacts of US2 and the BNSF railway on black bear mortality, particularly considering the increasing traffic volume on US2 due to surging visitation rates to Glacier [65, 66].

We did not find an effect of the transportation corridor on the movement of female bears in our models. However, evidence to the contrary exists based on high resolution movement data in other ecosystems [71–73, 91, 116, 126]. Only 8 females of 79 with spatial recaptures (10.1%) were recaptured within one sigma (1.7 km) of US2, and some sections along US2 had relatively fewer traps. Additionally, most females were spatially recaptured at short distances, most less than 1 km apart (Table 2, Fig. 2). It is possible that because females moved less and had small home ranges relative to the trap density near US2 that we were not able to detect effects of the corridor on female space use at the resolution of our cost space (250 m). In contrast, 21 of 114 (18.4%) spatially recaptured male bears were detected within one sigma (2.8 km for males) of US2, and capture–recapture data reflected more movement and greater distances moved over varied parts of the landscape (Table 2, Fig. 2). Other studies have found that female bears can exhibit negative density-dependence in which they contract their home range in high density areas because these areas are high quality habitat and/or support a higher number of territorial conspecifics, thus increasing the cost of moving greater distances from a natal area [48, 127–129]. This could be the case in Glacier because our model results showed that density increased with increasing forest cover and most of the region is dense forest habitat. Black bear densities are high in Glacier relative to other parts of the northern Rockies [90, 130].

The SCR approach to estimating landscape resistance to movement uses movement data that are temporally coarse, particularly compared to modern GPS/VHF telemetry data. With data that are temporally coarse (e.g., hair deposited sometime within two weeks) and if SCR data are sparse, uncertainty in the movement paths used to estimate resistance increases [37, 59]. Our dataset was large with many spatial recaptures and a high ratio of spatial to simple (non-spatial) recaptures to inform model parameters, but this is not typical among many SCR studies. SCR monitoring that results in few spatial recaptures of a target species due to low densities or sampling design will likely fail to reliably estimate resistance. However, if any telemetry data are available, integration of an explicit movement model via telemetry data with the SCR ecological distance model provides a path to improve the accuracy and precision of SCR resistance parameter estimates in all cases [59]. We also encountered computational limitations in applying the SCR ecological distance model to a large dataset across a large study area at a 250 m resolution. The SCR ecological distance model cannot currently be parallelized, and our models with multiple cost covariates on resistance required more than ten days to fit on a standard desktop computer and would not fit at all without truncating possible activity center locations to a reasonable distance from detections (i.e., using a ‘trimS’ value in oSCR).

The association of black bear movement with drainages and riparian vegetation cover likely reflects that bears in Glacier move through valleys more often than they cross mountainous terrain, and that riparian or mesic zones within mixed forests often have a greater abundance of food resources compared to other ecological zones [79, 131–133]. Studies from other North American populations also found GPS-tracked black bears selected for densely vegetated forest and riparian areas more than expected based on availability and moved slower in undisturbed forests compared to disturbed habitats [58, 102, 134, 135].

High use zones we identified generally align with previous expert-based efforts to find potential crossing zones based on field surveys of wildlife trails and wildlife-vehicle collision data for US-2, which also identified the areas from mile post 179–184, 154–155, and the zone from mile post 173–174 as potential wildlife crossing zones though these data may mostly reflect ungulates [80, 108]. However, our models also identified the area around mile post 154 just east of West Glacier as a potential DWC hotspot for black bears, and this location has not previously been considered a potential crossing zone. Because bears use drainages, widening existing culverts and riparian habitat enhancements surrounding culverts that coincide with high DWC areas could be a relatively efficient starting point to enhance connectivity for bears.

Our density estimates are consistent with previously reported densities of black bears that are sympatric with grizzlies in northern Idaho and northwest Montana (34.4–45.0 bears/100 km^2^, [131, 132, 136] and previous estimates for Glacier National Park and the Blackfeet Reservation (25 bears/100 km^2^, 95% CI 9–32, [130]). The support we found for non-homogenous density models and density increasing with increasing forest cover matches several studies estimating black bear densities in rural and natural landscapes [58, 77, 116, 132, 137]. Because the density of male bears decreased with increasing grizzly bear density, male density estimates were generally higher outside of or near the border of Glacier National Park than inside. This suggests competitive interactions between the two ursids which have been discussed in Stetz et al. [68], and highlights the importance of large, contiguous protected area networks for supporting sympatric populations of black and grizzly bears [138].

The predictive surfaces we generated are static and representative of the black bear population at a single point in time (2004). Wildfires and forest encroachment have shifted canopy cover in parts of the park since 2004, which has likely shifted bear distributions in these areas and may change patterns of density-weighted connectivity on the landscape. For example, in 2015, the Reynolds Creek Fire burned 19.5 km^2^ of forest along St. Mary Lake on the east side of the park and the Thompson Fire burned over 68 km^2^ near Nyack Creek in south-central Glacier. Additionally, traffic volume on US2 nearly doubled between 2001 and 2013, and thus the identified DWC hotspots along US2 may also have shifted since 2004 [64–66]. However, a benefit of the SCR approach is that if SCR monitoring continues over time, changes in demography and connectivity can be readily detected. The SCR approach to estimating population connectivity may be particularly useful if population density is spatially structured and the ecological variables shaping variation in density differ from the variables influencing movement. For recovering or declining wildlife populations, monitoring using a SCR design highlights not only where density is decreasing or increasing, but also the relationship between these demographic changes and changes in connectivity. Such scenarios are increasingly likely for wildlife populations given ongoing rapid land development in rural areas and climate change [64].

Future work could assess how DWC hotpots might shift seasonally or annually. It could also be valuable to validate results with remote camera surveys along the corridor comparing the amount of black bear use in high DWC vs. low DWC areas, or  with telemetry studies paired with experimental reduced speed zones or traffic limits on US2 during active periods for bears [139]. We also recognize that black bears are habitat generalists and evidence for their effective role as connectivity surrogates for habitat specialist species is limited [140]. As such, mitigation efforts based on black bear data alone may not improve connectivity for other species of concern to wildlife managers in Glacier such as grizzly bears or bighorn sheep (*Ovis canadensis*).

## Conclusions

Our application of contemporary SCR modeling techniques to archived data revealed new insights about black bear space use in the Crown of the Continent Ecosystem. Our study is one of few applications of SCR ecological distance models to estimate sex-specific resistance values at a reasonably fine resolution across a large area and we offer an example of how this approach can be used to prioritize locations where interventions to improve population connectivity may be most effective. We found that the Middle Fork Flathead River-US2-BNSF railroad transportation corridor strongly increased resistance to movement presenting a barrier to movement connectivity for male black bears, but that drainages, valley bottoms, and riparian vegetation decreased estimates of landscape resistance to movement for both male and female bears. For black bears in Glacier and surrounding landscapes, consideration of both vegetation and valley topography could inform the placement of underpasses along the transportation corridor in areas characterized by both high population density and potential connectivity. Contrary to the assumptions of some other connectivity modeling approaches, we found that not all locations with high potential connectivity were used by a high number of bears as the density-weighed connectivity surfaces we calculated highlighted areas important for population connectivity that were distinct from areas with high potential connectivity. Overall, our study demonstrates that the SCR ecological distance model can be applied to empirical data to create biologically realistic, spatially explicit predictions to support connectivity planning.

### Supplementary Information

**Additional file 1.** Details of spatial covariate data used for parameter estimation in spatial capture-recapture models for male and female black bears in Glacier National Park.

**Additional file 2.** Detection probabilities and comprehensive model selection results for multi-stage spatial capture-recapture ecological distance models for male and female black bears in Glacier National Park.

**Additional file 3.** R code to calculate density-weighted connectivity.

## Data Availability

The datasets used and analyzed during the study are published at https://doi.org/10.5066/P9V1HMLX.

## Abbreviations

SCR: Spatial capture-recapture
BNSF Railroad: Burlington Northern Santa Fe Railroad
US2: US highway 2
PC: Potential connectivity
DWC: Density-weighted connectivity
