# Supplementary material for: Evaluating density-weighted connectivity of black bears (Ursus americanus) in Glacier National Park with spatial capture–recapture models
Source: Mov Ecol. 2024 Jan 23;12:8. doi: 10.1186/s40462-023-00445-7 (PMC11334611; doi:10.1186/s40462-023-00445-7)
Supplement: Supplementary file 1 — Additional file 1. Details of spatial covariate data used for parameter estimation in spatial capture-recapture models for male and female black bears in Glacier National Park. [file 40462_2023_445_MOESM1_ESM.docx]

**Evaluating density-weighted connectivity of black bears (*Ursus americanus*) in Glacier National Park with spatial capture-recapture models**

*Movement Ecology* DOI: 10.1186/s40462-023-00445-7

Sarah L. Carroll^1^, Greta M. Schmidt, John S. Waller, Tabitha A. Graves

**^1^Corresponding Author:** Sarah L. Carroll, Graduate Degree Program in Ecology, Colorado State University, Fort Collins, CO 80523 [slcarrol@colostate.edu](mailto:slcarrol@colostate.edu), ORC-ID: 0000-0002-5391-7627

**Additional file 1. Details of spatial covariate data used for parameter estimation in spatial capture recapture models for male and female black bears in Glacier National Park [1].**

Any use of trade, firm, or product names is for descriptive purposes only and does not imply endorsement by the U.S. Government.

We calculated percent forest cover from 30m [USGS LANDFIRE](https://landfire.gov/getdata.php) Existing Vegetation Types (2004) data to represent variation in vegetation structure as closed canopy forest is the dominant landcover across 65% of the study area (Table S1; [2]). Because LANDFIRE products cover only the United States, we used percent forest cover from land cover layer data based on satellite imagery (30m) from the year 2000, covering the Canadian portion of the study area created by the Great Northern Landscape Conservation Cooperative and the Crown Managers Partnership (CMP) available at [Sciencebase](https://www.sciencebase.gov/catalog/item/565f3b40e4b071e7ea54453e) [3]. We also extracted riparian/mesic vegetation cover classes (riparian, wetlands, swamps) and deciduous cover (predominantly aspen) from the LANDFIRE vegetation data in 2004 and the CMP land use/land cover map with data for Canada. We obtained a raster for the study area representing estimated grizzly bear densities based on a SCR study conducted across northern Montana in 2004 from the authors of [4].

Over 864 km^2^ of the study area burned in wildfires in 2001 and 2003. We hypothesized that recent high intensity fires could negatively impact bear densities and so we extracted fire perimeters and burn severity data categorized as low, medium, or high from the [MTBS](https://www.mtbs.gov/) database [5]. We calculated the mean and standard deviation of total terrain curvature, derived from a National Elevation Dataset Digital Elevation Model (DEM) to account for variation in terrain complexity and differentiate continuous slopes from broken slopes [6,7]. We hypothesized that food availability would be patchy across the landscape and would influence detection probability and bear density where foods are less abundant, particularly early in the season when snow cover persists. To account for snow cover, we created a binary (snow on/off), trap-specific snow cover variable using the [Normalized Difference Snow Index](https://www.usgs.gov/landsat-missions/normalized-difference-snow-index) (NDSI) from Landsat 7 [8]. We calculated the variance in the start of spring (‘Greenup’) derived from MODIS Global Vegetation Phenology ([MCD12Q2.006 Land Cover Dynamics](https://lpdaac.usgs.gov/products/mcd12q2v006/)) effects of spatial heterogeneity in the timing of vegetation emergence on bear densities (Table S1,[9]). We hypothesized that areas with greater temporal variation in the high-protein ‘green-up’ phase of vegetation would provide more diverse and persistent vegetative food sources throughout the summer and thus potentially support a greater number of bears.

The study area is inter-jurisdictional and the strength of bear protection regulations that prevent bears from obtaining human foods and waste and minimize human impacts on bears is variable between jurisdictions [10]. Stronger protections could reduce bear mortalities as bears that learn to seek human foods are often destroyed and thus influence bear densities. We ranked regions of the study area for bear security based on the expert opinion of regional bear managers following Graves et al. (2011) [10]. Glacier National Park was assigned the highest protection (=10), USFS land = 7, other state and federal agencies and corporate forestry land (semi-protected) = 3, and private land = 1 [10]. In addition, human presence and land-use intensity can have negative effects on bear space use [11]. Alternatively, depending on bear protections, developed areas can serve as ecological traps that provide high caloric food rewards to bears, resulting in increased use by bears and subsequently increased human-caused mortality, [12,13]. We calculated paved road density (km/4km^2^) to index human land-use intensity because bears may select against areas of high road density [14,15] and paved road density was strongly correlated with developed landcover and human population density in our study area [16].

**
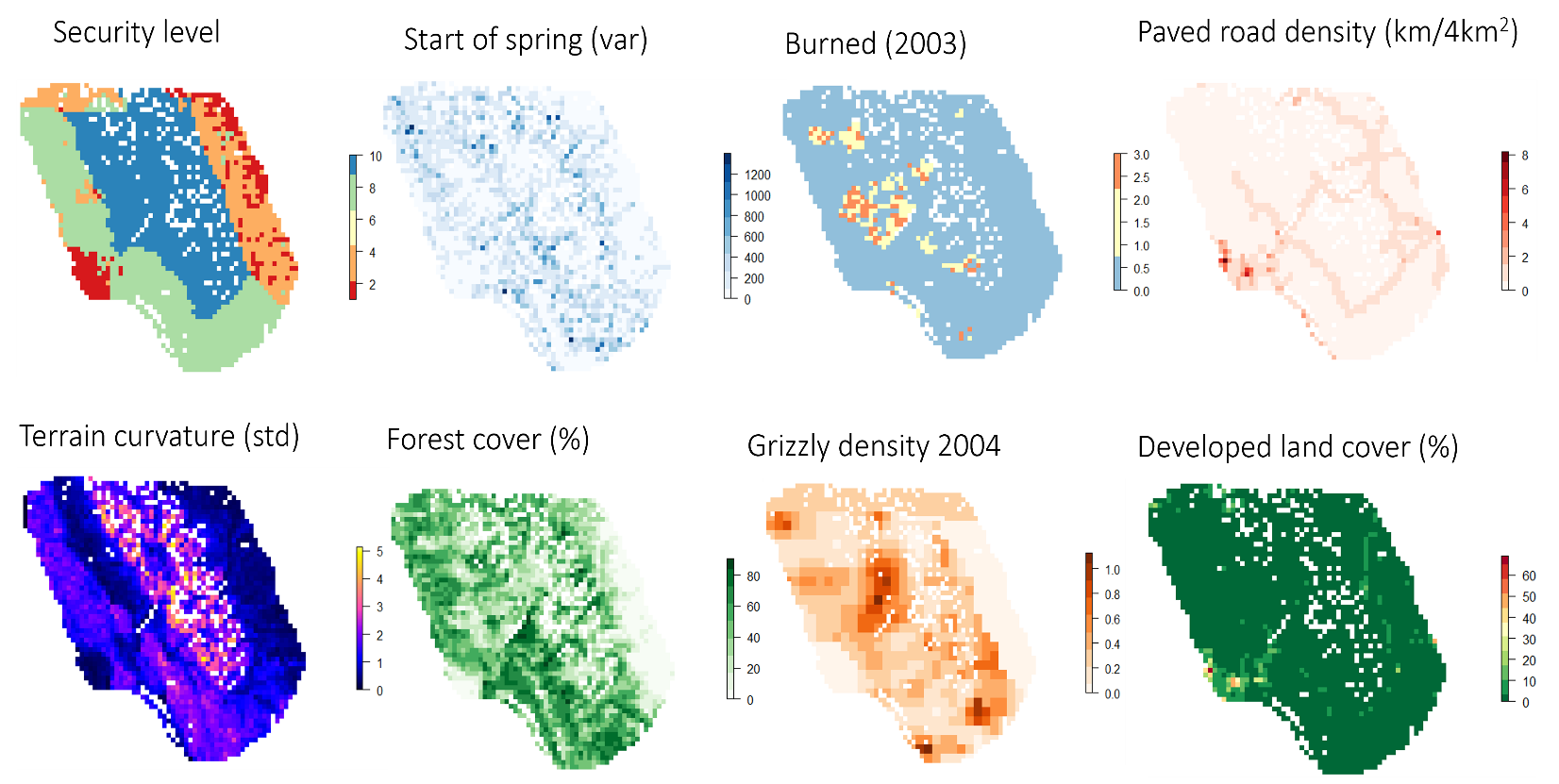
**

Figure S2. Mapped covariates (2km^2^) hypothesized to influence variation in black bear densities in Glacier National Park.

Table S1. Covariates hypothesized to influence SCR model estimates of density and detection probabilities for black bears with the hypothesized effect stated in terms of the hypothesized effect with increasing values of the covariate. The movement scaling parameter (σ) is not included because we modeled males and females separately and had no other individual-level covariates.

| **SCR model Parameter** | **Covariate** | **Hypothesized effect** | | **Evidence** | **Data Source** |
| --- | --- | --- | --- | --- | --- |
| ***D*** (density) | percent forest cover | positive | [17,18] | | [2]; CMP |
|  | variation in start of spring | positive | [19] | | [9] |
|  | terrain curvature | negative |  |  |  |
|  | recent fires | negative | ″ | | [5] |
|  | grizzly bear density (2004 estimate) | negative | [20] | | [4] |
|  | burn severity    habitat security/ land jurisdiction | negative    positive | [21]  [10,20] | | [5] |
| ***p*0** (base detection rate) | max. snow cover | negative | [17,18] | | [8] |
|  | percent forest cover | positive |  |  | [2]; CMP |
|  | terrain curvature | negative | [19] | | [7] |
|  | Julian day | either way | [19] | |  |
|  | rub behavior | positive |  |  |  |
|  | sampling effort | positive | [4,22]  ″ | |  |
|  | detector type | positive effect of  baited hair snares |  |  |  |


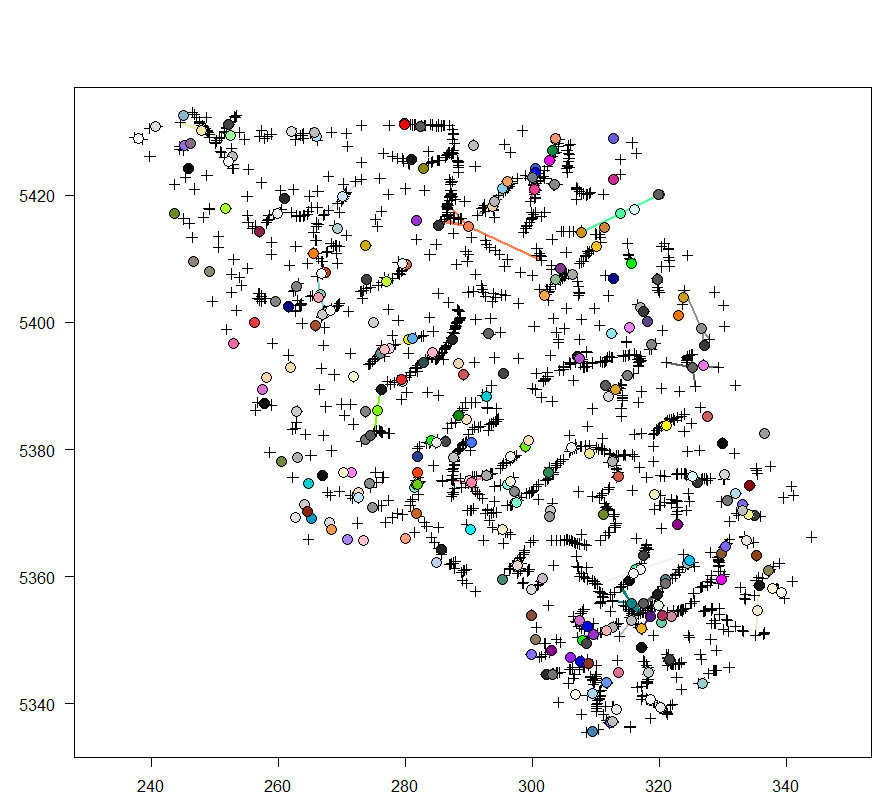

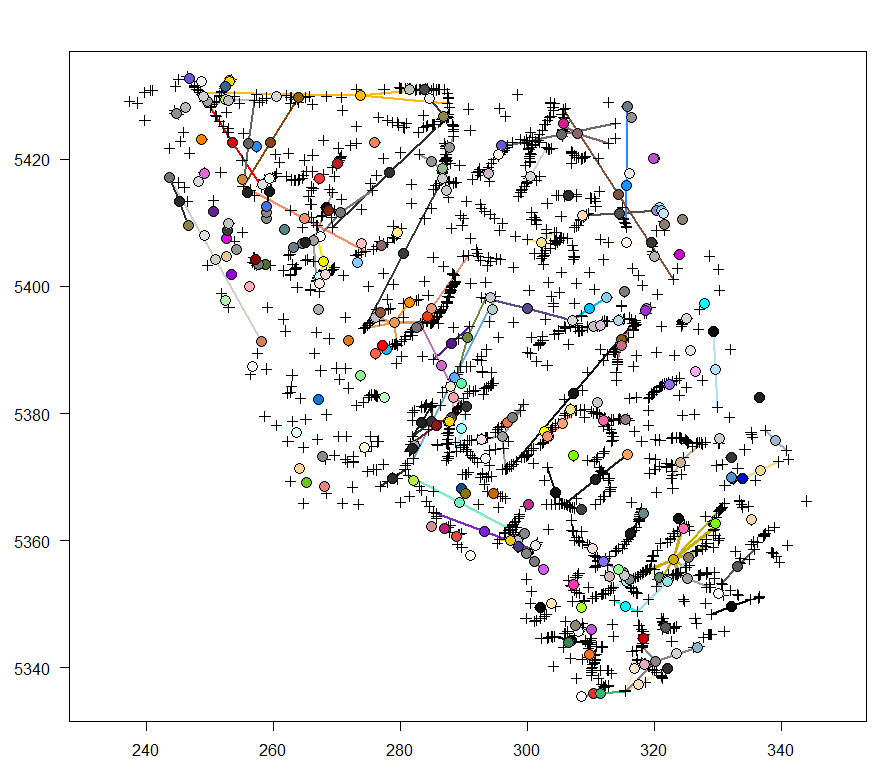


MALES

FEMALES

Figure S3. Spider plot depicting all SCR black bear detections (color dots) and traps (+) including spatial recaptures (movements among traps by individual) are represented with lines during a SCR study in Glacier National Park, Montana, USA in 2004.

**References**

1. Carroll S, Schmidt G, Waller J, Graves T. Black bear spatial capture-recapture dataset in and near Glacier National Park, Montana, USA, 2004. 2023.

2. LANDFIRE. Existing Vegetation Type Layer, LANDFIRE 1.0.5 [Internet]. U.S. Department of the Interior, Geological Survey, and U.S. Department of Agriculture. 2003. Available from: http://www.landfire/viewer

3. Sexton E. Land Use & Land Cover in the Crown of Continent Ecosystem c2000.2015. https://www.sciencebase.gov/catalog/item/51102e04e4b048b5cead853b

4. Kendall KC, Stetz JB, Boulanger J, Macleod AC, Paetkau D, White GC. Demography and Genetic Structure of a Recovering Grizzly Bear Population. Journal of Wildlife Management [Internet]. 2009;73:3–17. doi/abs/10.2193/2008-330

5. MTBS Project UDSA Forest Service/U.S. Geological Survey. MTBS Data Access: Fire Level Geospatial Data. 2010.

6. Erin Ironside K, J. Mattson D, Arundel T, Theimer T, Holton B, Peters M, et al. Geomorphometry in Landscape Ecology: Issues of Scale, Physiography, and Application. Environment and Ecology Research. 2018;6:397–412.

7. Gesch D, Oimoen M, Greenlee S, Nelson C, Steuck M, Tyler D. The national elevation dataset. Photogramm Eng Remote Sensing. 2002;68:5–32.

8. Riggs GA, Hall DK, Román MO. MODIS snow products collection 6 user guide. National Snow and Ice Data Center: Boulder, CO, USA. 2015;66.

9. Friedl M, Sulla-Menashe D. MCD12Q1 MODIS/Terra+ Aqua Land Cover Type Yearly L3 Global 500m SIN Grid V006, NASA EOSDIS Land Processes DAAC [data set].

10. Graves TA, Kendall KC, Royle JA, Stetz JB, Macleod AC. Linking landscape characteristics to local grizzly bear abundance using multiple detection methods in a hierarchical model. Anim Conserv. 2011;14:652–64.

11. Laufenberg JS, Johnson HE, Doherty PF, Breck SW. Compounding effects of human development and a natural food shortage on a black bear population along a human development-wildland interface. Biol Conserv. 2018;224:188–98.

12. Lamb CT, Mowat G, McLellan BN, Nielsen SE, Boutin S. Forbidden fruit: human settlement and abundant fruit create an ecological trap for an apex omnivore. Journal of Animal Ecology. 2017;86:55–65.

13. Johnson HE, Lewis DL, Breck SW. Individual and population fitness consequences associated with large carnivore use of residential development. Ecosphere. 2020;11.

14. Proctor MF, McLellan BN, Stenhouse GB, Mowat G, Lamb CT, Boyce MS. Effects of roads and motorized human access on grizzly bear populations in British Columbia and Alberta, Canada. Ursus. 2020;2019:16–39.

15. Simek SL, Belant JL, Fan Z, Young BW, Leopold BD, Fleming J, et al. Source populations and roads affect American black bear recolonization. Eur J Wildl Res. 2015;61:583–90.

16. Roever CL, Boyce MS, Stenhouse GB. Grizzly bear movements relative to roads: Application of step selection functions. Ecography. 2010;33:1113–22.

17. Sun CC, Fuller AK, Hare MP, Hurst JE. Evaluating population expansion of black bears using spatial capture‐recapture. J Wildl Manage. 2017;81:814–23.

18. Evans MJ, Rittenhouse TAG, Hawley JE, Rego PW. Black bear recolonization patterns in a human-dominated landscape vary based on housing: New insights from spatially explicit density models. Landsc Urban Plan. 2017;162:13–24.

19. Kendall KC, Graves TA, Royle JA, Macleod AC, McKelvey KS, Boulanger J, et al. Using bear rub data and spatial capture-recapture models to estimate trend in a brown bear population. Sci Rep. 2019;9:1–11.

20. Stetz JB, Mitchell MS, Kendall KC. Using spatially-explicit capture–recapture models to explain variation in seasonal density patterns of sympatric ursids. Ecography. 2019;42:237–48.

21. Cunningham SC, Ballard WB, Monroe LM, Rabe MJ, Bristow KD. Black bear habitat use in burned and unburned areas, central Arizona. Wildl Soc Bull. 2003;786–92.

22. Stetz JB, Kendall KC, Macleod AC. Black bear density in glacier national park, Montana. Wildl Soc Bull. 2014;38:60–70.
