# Supplementary material for: Evaluating density-weighted connectivity of black bears (Ursus americanus) in Glacier National Park with spatial capture–recapture models
Source: Mov Ecol. 2024 Jan 23;12:8. doi: 10.1186/s40462-023-00445-7 (PMC11334611; doi:10.1186/s40462-023-00445-7)
Supplement: Supplementary file 2 — Additional file 2. Detection probabilities and comprehensive model selection results for multi-stage spatial capture-recapture ecological distance models for male and female black bears in Glacier National Park. [file 40462_2023_445_MOESM2_ESM.docx]

**Evaluating density-weighted connectivity of black bears (*Ursus americanus*) in Glacier National Park with spatial capture-recapture models**

*Movement Ecology* DOI: 10.1186/s40462-023-00445-7

Sarah L. Carroll^1^, Greta M. Schmidt, John S. Waller, Tabitha A. Graves

**^1^Corresponding Author:** Sarah L. Carroll, Graduate Degree Program in Ecology, Colorado State University, Fort Collins, CO 80523 [slcarrol@colostate.edu](mailto:slcarrol@colostate.edu), ORC-ID: 0000-0002-5391-7627

**Additional file 2. Detection Probabilities and Comprehensive model selection results for multi-stage SCR ecological distance models for male and female black bears in Glacier National Park [1].**

Any use of trade, firm, or product names is for descriptive purposes only and does not imply endorsement by the U.S. Government.

[1]. Carroll SL, Schmidt G, Waller J, Graves TA. Black bear spatial capture-recapture dataset in and near Glacier National Park, Montana, USA, 2004: U.S. Geological Survey Data Release. 2023. <https://doi.org/10.5066/P9V1HMLX>

**
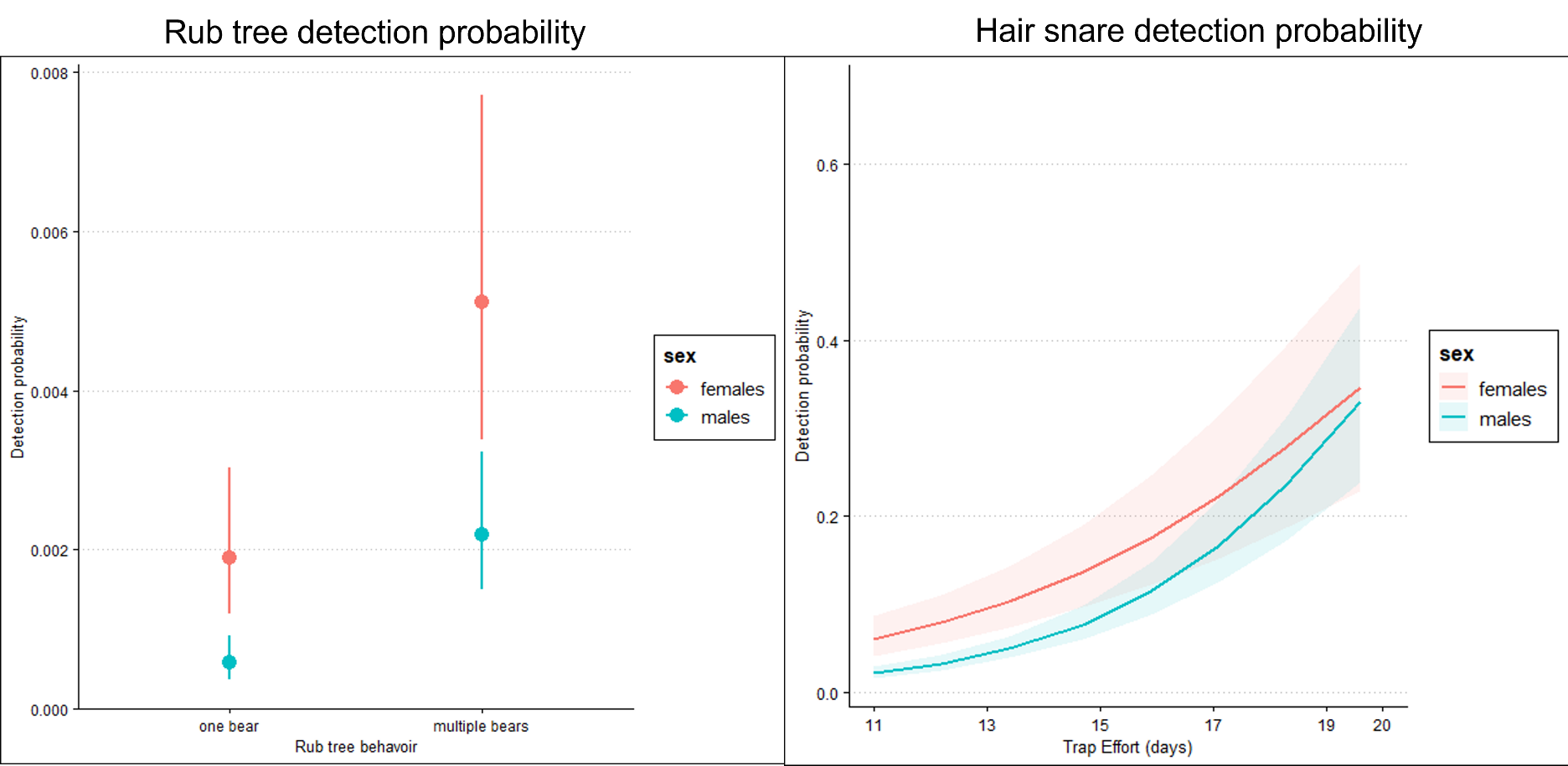
**

Figure S1. Predicted detection probabilities for male and female black bears showing covariate effects of trap type, rubbing behavior, and trap effort on detection probabilities for rub trees (left) and hair snares (right) calculated from the most supported SCR ecological distance models.

Table S1. Model selection table for all sub-model plausible combinations (42) of density and detection parameters models for **male** black bears. Note that top models for density included additive combinations of forest cover, griz density, and curv (7 models) and the top plausible combination of detection covariates consisted of those described in models 29, 30, 19, 3, and 14 leading to 7*6= 42 combinations.

| Model^a^  (males) | *D* | *p0* | logL | K | AIC | ∆ AIC | Weight (wi) | CumWt |
| --- | --- | --- | --- | --- | --- | --- | --- | --- |
| 29 | ~forest cover + griz density | ~Hair + BE + forest cover + Jul + Jul2 + ER + EH + curv | 1500.74 | 13 | 3027.47 | 0.00 | 0.26 | 0.26 |
| 5 | ~forest cover | ~Hair + BE + forest cover + Jul + Jul2 + ER + EH + curv | 1502.18 | 12 | 3028.36 | 0.89 | 0.16 | 0.42 |
| 23 | ~forest cover + curv + griz density | ~Hair + BE + forest cover + Jul + Jul2 + ER + EH + curv | 1500.34 | 14 | 3028.68 | 1.21 | 0.14 | 0.56 |
| 30 | ~forest cover + griz density | ~Hair + BE + forest cover + Jul + Jul2 + ER + EH + curv + snow | 1500.65 | 14 | 3029.31 | 1.83 | 0.10 | 0.66 |
| 17 | ~forest cover + curv | ~Hair + BE + forest cover + Jul + Jul2 + ER + EH + curv | 1501.82 | 13 | 3029.65 | 2.17 | 0.09 | 0.75 |
| 6 | ~forest cover | ~Hair + BE + forest cover + Jul + Jul2 + ER + EH + curv + snow | 1502.08 | 13 | 3030.16 | 2.68 | 0.07 | 0.82 |
| 24 | ~forest cover + curv + griz density | ~Hair + BE + forest cover + Jul + Jul2 + ER + EH + curv + snow | 1500.26 | 15 | 3030.52 | 3.05 | 0.06 | 0.87 |
| 18 | ~forest cover + curv | ~Hair + BE + forest cover + Jul + Jul2 + ER + EH + curv + snow | 1501.72 | 14 | 3031.45 | 3.97 | 0.04 | 0.91 |
| 35 | ~griz density | ~Hair + BE + forest cover + Jul + Jul2 + ER + EH + curv | 1503.77 | 12 | 3031.54 | 4.07 | 0.03 | 0.94 |
| 41 | ~griz density + curv | ~Hair + BE + forest cover + Jul + Jul2 + ER + EH + curv | 1503.24 | 13 | 3032.48 | 5.01 | 0.02 | 0.96 |
| 36 | ~griz density | ~Hair + BE + forest cover + Jul + Jul2 + ER + EH + curv + snow | 1503.70 | 13 | 3033.40 | 5.93 | 0.01 | 0.98 |
| 42 | ~griz density + curv | ~Hair + BE + forest cover + Jul + Jul2 + ER + EH + curv + snow | 1503.18 | 14 | 3034.35 | 6.88 | 0.01 | 0.98 |
| 11 | ~curv | ~Hair + BE + forest cover + Jul + Jul2 + ER + EH + curv | 1505.29 | 12 | 3034.58 | 7.11 | 0.01 | 0.99 |
| 12 | ~curv | ~Hair + BE + forest cover + Jul + Jul2 + ER + EH + curv + snow | 1505.20 | 13 | 3036.41 | 8.94 | 0.00 | 0.99 |
| 19 | ~forest cover + curv + griz density | ~Hair + BE + forest cover + Jul + Jul2 + ER + EH | 1505.77 | 13 | 3037.55 | 10.07 | 0.00 | 1.00 |
| 13 | ~forest cover + curv | ~Hair + BE + forest cover + Jul + Jul2 + ER + EH | 1507.47 | 12 | 3038.95 | 11.48 | 0.00 | 1.00 |
| 37 | ~griz density + curv | ~Hair + BE + forest cover + Jul + Jul2 + ER + EH | 1507.49 | 12 | 3038.98 | 11.51 | 0.00 | 1.00 |
| 22 | ~forest cover + curv + griz density | ~Hair + BE + forest cover + Jul + Jul2 + ER + EH + snow | 1505.70 | 14 | 3039.40 | 11.92 | 0.00 | 1.00 |
| 16 | ~forest cover + curv | ~Hair + BE + forest cover + Jul + Jul2 + ER + EH + snow | 1507.38 | 13 | 3040.76 | 13.28 | 0.00 | 1.00 |
| 40 | ~griz density + curv | ~Hair + BE + forest cover + Jul + Jul2 + ER + EH + snow | 1507.43 | 13 | 3040.86 | 13.38 | 0.00 | 1.00 |
| 25 | ~forest cover + griz density | ~Hair + BE + forest cover + Jul + Jul2 + ER + EH | 1508.46 | 12 | 3040.91 | 13.44 | 0.00 | 1.00 |
| 7 | ~curv | ~Hair + BE + forest cover + Jul + Jul2 + ER + EH | 1509.67 | 11 | 3041.33 | 13.86 | 0.00 | 1.00 |
| 31 | ~griz density | ~Hair + BE + forest cover + Jul + Jul2 + ER + EH | 1510.01 | 11 | 3042.02 | 14.55 | 0.00 | 1.00 |
| 1 | ~forest cover | ~Hair + BE + forest cover + Jul + Jul2 + ER + EH | 1510.07 | 11 | 3042.13 | 14.66 | 0.00 | 1.00 |
| 28 | ~forest cover + griz density | ~Hair + BE + forest cover + Jul + Jul2 + ER + EH + snow | 1508.38 | 13 | 3042.75 | 15.28 | 0.00 | 1.00 |
| 10 | ~curv | ~Hair + BE + forest cover + Jul + Jul2 + ER + EH + snow | 1509.58 | 12 | 3043.16 | 15.69 | 0.00 | 1.00 |
| 34 | ~griz density | ~Hair + BE + forest cover + Jul + Jul2 + ER + EH + snow | 1509.94 | 12 | 3043.88 | 16.41 | 0.00 | 1.00 |
| 4 | ~forest cover | ~Hair + BE + forest cover + Jul + Jul2 + ER + EH + snow | 1509.97 | 12 | 3043.93 | 16.46 | 0.00 | 1.00 |
| 3 | ~forest cover | ~Hair + BE + forest cover + Jul + ER + EH + curv | 1544.09 | 11 | 3110.19 | 82.71 | 0.00 | 1.00 |
| 27 | ~forest cover + griz density | ~Hair + BE + forest cover + Jul + ER + EH + curv | 1543.38 | 12 | 3110.77 | 83.30 | 0.00 | 1.00 |
| 15 | ~forest cover + curv | ~Hair + BE + forest cover + Jul + ER + EH + curv | 1543.54 | 12 | 3111.07 | 83.60 | 0.00 | 1.00 |
| 21 | ~forest cover + curv + griz density | ~Hair + BE + forest cover + Jul + ER + EH + curv | 1542.78 | 13 | 3111.55 | 84.08 | 0.00 | 1.00 |
| 33 | ~griz density | ~Hair + BE + forest cover + Jul + ER + EH + curv | 1546.53 | 11 | 3115.06 | 87.58 | 0.00 | 1.00 |
| 39 | ~griz density + curv | ~Hair + BE + forest cover + Jul + ER + EH + curv | 1545.73 | 12 | 3115.47 | 87.99 | 0.00 | 1.00 |
| 9 | ~curv | ~Hair + BE + forest cover + Jul + ER + EH + curv | 1546.85 | 11 | 3115.70 | 88.22 | 0.00 | 1.00 |
| 14 | ~forest cover + curv | ~Hair + BE + forest cover + Jul + ER + EH + snow | 1548.98 | 12 | 3121.95 | 94.48 | 0.00 | 1.00 |
| 20 | ~forest cover + curv + griz density | ~Hair + BE + forest cover + Jul + ER + EH + snow | 1548.05 | 13 | 3122.10 | 94.63 | 0.00 | 1.00 |
| 38 | ~griz density + curv | ~Hair + BE + forest cover + Jul + ER + EH + snow | 1549.84 | 12 | 3123.69 | 96.21 | 0.00 | 1.00 |
| 8 | ~curv | ~Hair + BE + forest cover + Jul + ER + EH + snow | 1551.07 | 11 | 3124.13 | 96.66 | 0.00 | 1.00 |
| 2 | ~forest cover | ~Hair + BE + forest cover + Jul + ER + EH + snow | 1552.17 | 11 | 3126.33 | 98.86 | 0.00 | 1.00 |
| 26 | ~forest cover + griz density | ~Hair + BE + forest cover + Jul + ER + EH + snow | 1551.34 | 12 | 3126.68 | 99.21 | 0.00 | 1.00 |
| 32 | ~griz density | ~Hair + BE + forest cover + Jul + ER + EH + snow | 1552.99 | 11 | 3127.99 | 100.52 | 0.00 | 1.00 |

**^a^ Model notation:**

Hair: trap type is or is not a hair snare

BE: rub behavior effect

Jul: Julian day, linear effect of season

Jul2: Julian day squared, quadratic effect of season

ER: bear rub trap effort, number of days trap open during each occasion

EH: hair snare trap effort, number of days trap open during each occasion

curv: standard deviation of terrain curvature

Snow: presence/absence of snow cover at trap on day of detection

griz density: estimated grizzly bear density in 2004 in each pixel

Table S2. Model selection table for all sub-model plausible combinations (12) of density and detection parameters models for **female** black bears. Note that the two top models for density were the null model and forest cover, and the top plausible combination of detection covariates consisted of those described in models 3, 5, 1, 11, 6, and 4, leading to 2*6= 12 combinations.

| Model  (females) | *D* | *p0* | logL | K | AIC | ∆ AIC | weight | CumWt |
| --- | --- | --- | --- | --- | --- | --- | --- | --- |
| 3 | ~forest cover | ~Hair + BE + forest cover + Jul + ER + EH + curv | 1068.91 | 11 | 2159.83 | 0.00 | 0.21 | 0.21 |
| 5 | ~forest cover | ~Hair + BE + forest cover + Jul + Jul2 + ER + EH + curv | 1068.05 | 12 | 2160.10 | 0.27 | 0.19 | 0.40 |
| 9 | ~1 | ~Hair + BE + forest cover + Jul + ER + EH + curv | 1070.65 | 10 | 2161.31 | 1.48 | 0.10 | 0.50 |
| 1 | ~forest cover | ~Hair + BE + forest cover + Jul + Jul2 + ER + EH | 1069.70 | 11 | 2161.39 | 1.57 | 0.10 | 0.60 |
| 11 | ~1 | ~Hair + BE + forest cover + Jul + Jul2 + ER + EH + curv | 1069.79 | 11 | 2161.57 | 1.75 | 0.09 | 0.68 |
| 7 | ~1 | ~Hair + BE + forest cover + Jul + Jul2 + ER + EH | 1070.99 | 10 | 2161.99 | 2.16 | 0.07 | 0.76 |
| 6 | ~forest cover | ~Hair + BE + forest cover + Jul + Jul2 + ER + EH + curv + snow | 1068.01 | 13 | 2162.03 | 2.20 | 0.07 | 0.83 |
| 2 | ~forest cover | ~Hair + BE + forest cover + Jul + ER + EH + snow | 1070.54 | 11 | 2163.08 | 3.25 | 0.04 | 0.87 |
| 4 | ~forest cover | ~Hair + BE + forest cover + Jul + Jul2 + ER + EH + snow | 1069.65 | 12 | 2163.30 | 3.47 | 0.04 | 0.91 |
| 12 | ~1 | ~Hair + BE + forest cover + Jul + Jul2 + ER + EH + curv + snow | 1069.75 | 12 | 2163.50 | 3.67 | 0.03 | 0.94 |
| 8 | ~1 | ~Hair + BE + forest cover + Jul + ER + EH + snow | 1071.84 | 10 | 2163.67 | 3.85 | 0.03 | 0.97 |
| 10 | ~1 | ~Hair + BE + forest cover + Jul + Jul2 + ER + EH + snow | 1070.94 | 11 | 2163.89 | 4.06 | 0.03 | 1.00 |

Table S3. Final candidate model selection table for ecological distance models (resistance to movement parameter; δ) for **male** black bears.

| Model^b^ (males) | *D* | *p*0 | asu (δ) | LogL | K | AIC | ∆ AIC | Weight (wi) | CumWt |
| --- | --- | --- | --- | --- | --- | --- | --- | --- | --- |
| 4 | ~forest cover + griz density | ~ best | ~ distance to drain + transport corridor + forest cover | 1485.69 | 16 | 3003.38 | 0.00 | 0.33 | 0.33 |
| 5 | ~forest cover + griz density | ~ best | ~ distance to drain + transport corridor + riparian | 1485.78 | 16 | \| 3003.56 \| 0.18 \| 0.30 \| 0.64 \| \| --- \| --- \| --- \| --- \| | 0.18 | 0.30 | 0.64 |
| 2 | ~forest cover + griz density | ~ best | ~ distance to drain + transport corridor | 1487.36 | 15 | 3004.72 | 1.34 | 0.17 | 0.81 |
| 6 | ~forest cover + griz density | ~ best | ~ transport corridor + forest cover + riparian | 1486.88 | 16 | 3005.77 | 2.38 | 0.10 | 0.91 |
| 3 | ~forest cover + griz density | ~ best | ~ transport corridor + forest cover | 1488.01 | 15 | 3006.01 | 2.63 | 0.09 | 1.00 |
| 1 | ~forest cover + griz density | ~ best | ~ distance to drain + forest cover | 1494.29 | 15 | 3018.59 | 15.21 | 0.00 | 1.00 |

**^b^ Model notation:**

forest cover: percent forest cover

griz density: estimated grizzly bear density in 2004 in each pixel

distance to drain: distance to nearest drainage

riparian: majority presence of riparian habitat in each pixel

transport corridor: the absence or presence of US2, the Burlington-Northern Santa Fe Railroad, or both in each pixel

Table S4. Univariate model selection table for ecological distance models (resistance to movement parameter; δ) for **female** black bears including the null resistance model. Note that because drainages and riparian cover had some collinearity, we proceeded with the binary drainages model for inference.

| Model^c^ (females) | *D* | *p*0 | asu (δ) | LogL | K | AIC | ∆ AIC | Weight (wi) | | CumWt |
| --- | --- | --- | --- | --- | --- | --- | --- | --- | --- | --- |
| 5 | ~forest cover | ~ best | ~ drain binary | 1065.61 | 12 | 2155.22 | 0.00 | 0.68 | 0.68 | |
| 10 | ~forest cover | ~ best | ~ riparian cover | 1067.41 | 12 | 2158.83 | 3.60 | 0.11 | 0.79 | |
| 16 | ~forest cover | ~ best | ~ **null** | 1068.91 | 11 | 2159.83 | 4.60 | 0.07 | 0.86 | |
| 9 | ~forest cover | ~ best | ~ distance to roads | 1067.97 | 12 | 2159.95 | 4.72 | 0.06 | 0.92 | |
| 6 | ~forest cover | ~ best | ~ distance to drain | 1069.16 | 12 | 2162.32 | 7.10 | 0.02 | 0.94 | |
| 14 | ~forest cover | ~ best | ~ distance to Hwy | 1069.83 | 12 | 2163.67 | 8.44 | 0.01 | 0.95 | |
| 8 | ~forest cover | ~ best | ~ GTSR | 1070.05 | 12 | 2164.11 | 8.88 | 0.01 | 0.96 | |
| 7 | ~forest cover | ~ best | ~ railway | 1070.19 | 12 | 2164.38 | 9.16 | 0.01 | 0.97 | |
| 3 | ~forest cover | ~ best | ~ aspen cover | 1070.27 | 12 | 2164.55 | 9.33 | 0.01 | 0.98 | |
| 4 | ~forest cover | ~ best | ~ transport corridor | 1070.31 | 12 | 2164.63 | 9.40 | 0.01 | 0.98 | |
| 2 | ~forest cover | ~ best | ~ roads binary | 1070.33 | 12 | 2164.66 | 9.43 | 0.01 | 0.99 | |
| 1 | ~forest cover | ~ best | ~ Hwy 2 | 1070.34 | 12 | 2164.68 | 9.46 | 0.01 | 0.99 | |
| 15 | ~forest cover | ~ best | ~ road density | 1070.34 | 12 | 2164.69 | 9.46 | 0.01 | 1.00 | |

**^c^ Model notation:**

Hwy 2: presence or absence of US2 in each pixel

Distance to Hwy: distance in km from each pixel to nearest pixel containing US2

GTSR: presence or absence of Going-to-the-Sun Road in each pixel

aspen cover: majority presence of deciduous, aspen dominant forest cover in each pixel

Table S5. Univariate model selection table for ecological distance models (resistance to movement parameter; δ) for **male** black bears including the null resistance model.

| Model (males) | *D* | *p*0 | asu (δ) | LogL | K | AIC | ∆ AIC | Weight (wi) | CumWt |
| --- | --- | --- | --- | --- | --- | --- | --- | --- | --- |
| 10 | ~forest cover + griz density | ~ best | ~ transport corridor | 1493.22 | 14 | 3014.44 | 0.00 | 0.40 | 0.40 |
| 9 | ~forest cover + griz density | ~ best | ~ railway | 1493.73 | 14 | 3015.45 | 1.01 | 0.24 | 0.64 |
| 12 | ~forest cover + griz density | ~ best | ~ Hwy 2 | 1493.80 | 14 | 3015.60 | 1.15 | 0.23 | 0.87 |
| 1 | ~forest cover + griz density | ~ best | ~ distance to drain | 1494.92 | 14 | 3017.84 | 3.39 | 0.07 | 0.94 |
| 7 | ~forest cover + griz density | ~ best | ~ drain binary | 1496.17 | 14 | 3020.35 | 5.90 | 0.02 | 0.97 |
| 15 | ~forest cover + griz density | ~ best | ~ forest cover | 1496.64 | 14 | 3021.28 | 6.83 | 0.01 | 0.98 |
| 3 | ~forest cover + griz density | ~ best | ~ riparian cover | 1497.01 | 14 | 3022.01 | 7.57 | 0.01 | 0.99 |
| 11 | ~forest cover + griz density | ~ best | ~ distance to Hwy | 1497.84 | 14 | 3023.68 | 9.24 | 0.00 | 0.99 |
| 6 | ~forest cover + griz density | ~ best | ~ road density | 1498.09 | 14 | 3024.18 | 9.73 | 0.00 | 0.99 |
| 8 | ~forest cover + griz density | ~ best | ~ roads binary | 1498.56 | 14 | 3025.11 | 10.67 | 0.00 | 1.00 |
| 13 | ~forest cover + griz density | ~ best | ~ aspen cover | 1499.36 | 14 | 3026.72 | 12.27 | 0.00 | 1.00 |
| 16 | ~forest cover + griz density | ~ best | ~ **null** | 1500.74 | 13 | 3027.47 | 13.03 | 0.00 | 1.00 |
| 5 | ~forest cover + griz density | ~ best | ~ distance to roads | 1499.86 | 14 | 3027.73 | 13.29 | 0.00 | 1.00 |
| 2 | ~forest cover + griz density | ~ best | ~ mean curvature | 1500.15 | 14 | 3028.30 | 13.86 | 0.00 | 1.00 |
| 14 | ~forest cover + griz density | ~ best | ~ GTSR | 1500.25 | 14 | 3028.50 | 14.06 | 0.00 | 1.00 |
| 4 | ~forest cover + griz density | ~ best | ~ SD curvature | 1500.25 | 14 | 3028.51 | 14.06 | 0.00 | 1.00 |
